# Current-induced self-switching of perpendicular magnetization in CoPt single layer

**DOI:** 10.1038/s41467-022-31167-w

**Published:** 2022-06-20

**Authors:** Liang Liu, Chenghang Zhou, Tieyang Zhao, Bingqing Yao, Jing Zhou, Xinyu Shu, Shaohai Chen, Shu Shi, Shibo Xi, Da Lan, Weinan Lin, Qidong Xie, Lizhu Ren, Zhaoyang Luo, Chao Sun, Ping Yang, Er-Jia Guo, Zhili Dong, Aurelien Manchon, Jingsheng Chen

**Affiliations:** 1 Department of Materials Science and Engineering, National University of Singapore, Singapore, 117575 Singapore; 2 School of Materials Science and Engineering, Nanyang Technological University, Singapore, 639798 Singapore; 3 Singapore Synchrotron Light Source (SSLS), National University of Singapore, 5 Research Link, Singapore, 117603 Singapore; 4 Department of Electrical and Computing Engineering, National University of Singapore, Singapore, 117583 Singapore; 5 Beijing National Laboratory for Condensed Matter Physics and Institute of Physics, Chinese Academy of Sciences, Beijing, 100190 China; 6 Aix-Marseille Univ, CNRS, CINaM, Marseille, France; 7 Suzhou Research Institute, National University of Singapore, Suzhou, 215123 China; 8 Chongqing Research Institute, National University of Singapore, Chongqing, 401120 China; 9 Institute of Material Research and Engineering, A*STAR, Singapore, 138634 Singapore; 10 Present Address: Institute of Sustainability for Chemicals, Energy and Environment, A*STAR (Agency for Science, Technology and Research), 1 Pesek Road, Jurong Island, Singapore

**Keywords:** Electronic and spintronic devices, Electrical and electronic engineering, Ferromagnetism, Spintronics, Magnetic properties and materials

## Abstract

All-electric switching of perpendicular magnetization is a prerequisite for the integration of fast, high-density, and low-power magnetic memories and magnetic logic devices into electric circuits. To date, the field-free spin-orbit torque (SOT) switching of perpendicular magnetization has been observed in SOT bilayer and trilayer systems through various asymmetric designs, which mainly aim to break the mirror symmetry. Here, we report that the perpendicular magnetization of Co_*x*_Pt_100-*x*_ single layers within a special composition range (20 < *x* < 56) can be deterministically switched by electrical current in the absence of external magnetic field. Specifically, the Co_30_Pt_70_ shows the largest out-of-plane effective field efficiency and best switching performance. We demonstrate that this unique property arises from the cooperation of two structural mechanisms: the low crystal symmetry property at the Co platelet/Pt interfaces and the composition gradient along the thickness direction. Compared with that in bilayers or trilayers, the field-free switching in Co_*x*_Pt_100-*x*_ single layer greatly simplifies the SOT structure and avoids additional asymmetric designs.

## Abstract (web summary)

One challenge for spin-based electronics is the controlled and reliable switching of magnetization without magnetic fields. Here, Liu et al investigate a variety of compositions of CoPt, and determine the specific composition to maximize switching performance, potentially simplifying device design.

## Introduction

Current-induced spin-orbit torque (SOT) has been attracting great interest owing to its effective manipulation of magnetization^1–3^. The SOT switching of perpendicular magnetization usually requires extra assistance from external magnetic field in conventional heavy metal/ferromagnet (HM/FM) bilayers. To achieve the field-free switching, many efforts have been made to break the mirror symmetry relative to the *xz* plane, assuming the current is along the *x* direction and the magnetization is along the *z* direction^4–14^. It can be realized by introducing a tilting effect (such as tilted structure^4,5^ or tilted anisotropy^6,7^) in the *yz* plane, a polar vector (such as electrical field^8^ or electric polarization) along the *y* direction, and an axis vector (such as interlayer coupling^9^, exchange bias^10–12^, stray field^13^, or additional magnetic layer^14^) along the *x* direction. The chiral symmetry breaking by a gradient of magnetic anisotropy or saturation magnetization can also induce field-free switching^15^. Different from the above approaches that require asymmetric designs in device/film structures, recent works utilized the crystal symmetry to engineer the field-free switching in the WTe_2_/FM bilayer^16–18^ and the CuPt/FM bilayer^19^. Although largely explored, the existing field-free switching in the above SOT bilayers/trilayers has some intrinsic disadvantages. First, the SOT (usually generated from an individual spin source layer) is an interface effect in nature, which constrains the thickness of the FM layer and limits the thermal stability of the memory cell. Second, the interfacial spin mixing may lead to a decrease in the switching efficiency. Therefore, it has been desirable to explore the current-induced field-free switching in a single ferromagnetic layer.

Previously, current-induced perpendicular magnetization switching in the single magnetic layer by bulk SOT has been demonstrated in diluted ferromagnets (GaMnAs^20^, GeMnTe^21^) with bulk inversion asymmetry, which however requires low temperature and external in-plane magnetic field. The SOT switching in a single layer of metallic ferromagnets (*L*1_0_-FePt^22,23^, CoTb^24^) by composition gradient or inner interface-induced inversion asymmetry brings the working temperature to 300 K, though the external in-plane magnetic field is still needed. Recently, the field-free SOT switching is realized by gradient-driven Dzyaloshinskii–Moriya interaction (DMI) induced by composition gradient in a CoTb layer^25^, where the deterministic switching starts with the domain nucleation at the edge of the sample due to the magnetization tilting.

Here we report on the observation of the current-induced field-free switching of perpendicular magnetization in Co_*x*_Pt_1-*x*_ single layers by combining the composition gradient-induced damping-like torque and the low crystal symmetry-induced “3m torque”. We show that the SOT in Co_*x*_Pt_1-*x*_ can be largely tuned by changing the composition ratio from *x* = 20 to *x* = 56, and the specific composition of Co_30_Pt_70_ was found to exhibit the best SOT switching performance. Based on the thickness and the composition dependences, we suggested that the field-free SOT switching in Co_30_Pt_70_ is closely related to the formation of Co platelets near the substrate.

## Results

### Structural and magnetic properties

In general, the Co_*x*_Pt_1-*x*_ alloys have different phases with varied compositions, including the chemically ordered *L*1_2_, *L*1_0_, *L*1_1_, and *D*0_19_ phases and the disordered A1 and A3 phases. Among all phases, the A1 disordered CoPt_3_ (with a certain *x* range) possesses low saturation magnetization (*M*_s_), which is desirable for energy-efficient magnetization switching. In our experiments, we deposited Co_30_Pt_70_ films with thicknesses varying from 6 to 12 nm on single-crystalline MgO (111) substrate at 300 °C (see the “Methods” section). The composition of Co_30_Pt_70_ was verified by energy-dispersive X-ray spectroscopy (EDS). It has been reported that the A1 disordered CoPt_3_ with a face-centered cubic (*fcc*) structure has perpendicular magnetic anisotropy (PMA) due to the excess of the in-plane Co–Co bonds with the formation of Co platelets on Pt-rich matrix during growth^26–29^. To confirm the bonding anisotropy in our Co_30_Pt_70_ films, we performed the X-ray absorption spectroscopy (XAFS) experiments and Supplementary Table S1 summarized the fitted parameters for different bonds in the in-plane directions and the out-of-plane directions (Supplementary Section 1). We found a dominated Co–Co in-plane pairing, which indicates the formation of Co platelets.

**Fig. 1:**
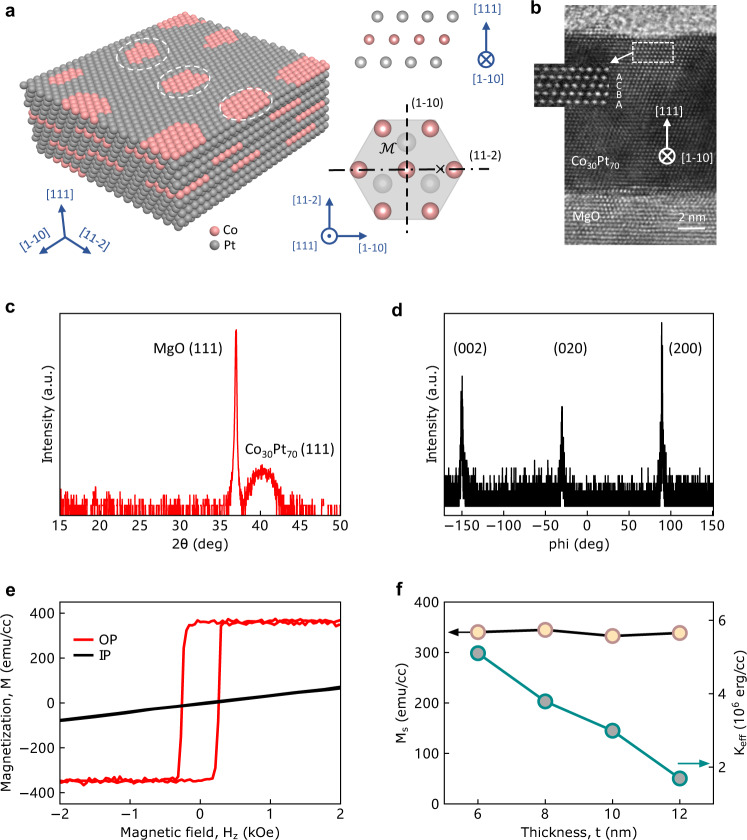
Structural and magnetic properties of Co_30_Pt_70_ thin film. **a** Left: the schematic illustration of Co_30_Pt_70_ with randomly located Co platelets (see white circles); right top: the side view of the Co platelet/Pt structure; right bottom, the mirror symmetry analysis of Co platelet/Pt, where [1-10] is defined as the low-symmetry axis and [11-2] as the high-symmetry axis, respectively. **b** High-resolution transmission electron microscopy (HR-TEM) image of Co_30_Pt_70_ with [1-10] pointing inward. The area within the dotted rectangle was amplified to present the stacking sequence (ABCABC…, from bottom to top) of Co_30_Pt_70_. **c** High-resolution X-ray diffraction (HR-XRD) pattern of Co_30_Pt_70_. **d** HR-XRD phi-scan pattern with Co_30_Pt_70_ (002) plane rotated along [111] axis. **e** Out-of-plane (OP) and in-plane (IP) magnetic hysteresis loops of the unpatterned Co_30_Pt_70_ thin film. **f** Thickness dependence of the saturation magnetization (*M*_s_) and effective perpendicular magnetic anisotropy energy (*K*_eff_).

The structure of Co_30_Pt_70_ with Co platelets is schematically illustrated on the left of Fig. 1a. The right top of Fig. 1a shows a side view of the Co platelet/Pt, which resembles the Co/Pt superlattice. The right bottom of Fig. 1a shows the top view of the Co platelet/Pt structure, where the in-plane mirror symmetry is broken with respect to the (11-2) plane (defined by [1-10] and [111]), while preserved with respect to the (1-10) plane (defined by [11-2] and [111]). According to the previous studies, the broken in-plane mirror symmetry would allow for an out-of-plane (OOP) spin-orbit torque^16^. The high-resolution transmission electron microscopy (HR-TEM) result of our Co_30_Pt_70_ in Fig. 1b shows a stacking sequence of ABCABC… from bottom to top, which is consistent with its *fcc* structure (see Supplementary Section 1 for more discussions). Figure 1c shows the high-resolution X-ray diffraction (HR-XRD) pattern of a 6 nm Co_30_Pt_70_ thin film with a (111) peak at 40.6°. The phi-scan pattern in Fig. 1d shows three peaks that indicate the three-fold rotational symmetry. The TEM and XRD results verified the *fcc* structure and the epitaxial growth of our Co_30_Pt_70_ film. Figure 1e shows the magnetic hysteresis (*M-H*) loops of the 6 nm Co_30_Pt_70_ thin film, where the squared out-of-plane *M-H* loop and the linear in-plane *M-H* relation indicate a good PMA. The *M*-*H* loops for Co_30_Pt_70_ with various thicknesses from 6 to 12 nm are shown in Supplementary Fig. S2. As summarized in Fig. 1f, the saturation magnetization (*M*_s_) remains almost constant with changing thickness, while the effective perpendicular magnetic anisotropy energy (*K*_eff_) shows a decreasing trend with thickness, indicating that the Co platelets mainly exist near the substrate.

### Current-induced SOT effective fields and field-free switching

**Fig. 2:**
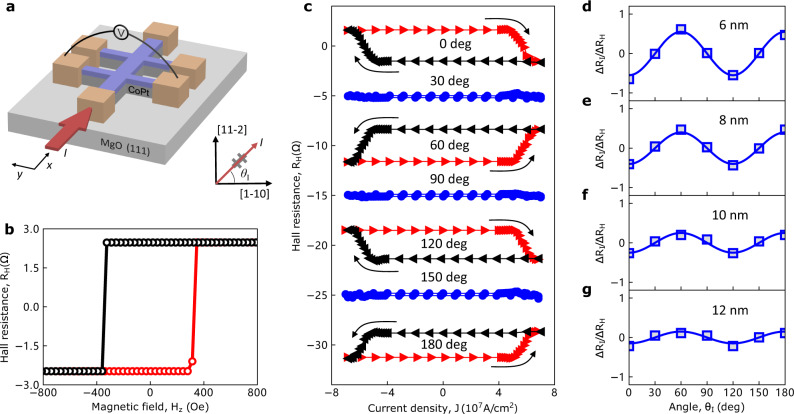
Field-free magnetization switching in Co_30_Pt_70_ single layers on MgO (111) substrate. **a** Schematic of the Co_30_Pt_70_ Hall bar device for electrical transport measurement. **b** Anomalous Hall effect of the 6 nm Co_30_Pt_70_ sample. **c** Current-induced field-free magnetization switching in Co_30_Pt_70_ for Hall bars with different *θ*_I_. The loops are manually shifted for better visualization. **d**–**g** *θ*_I_ dependence of Δ*R*_I_/Δ*R*_H_ for Co_30_Pt_70_ devices with different thicknesses (6, 8, 10, and 12 nm).

We patterned the Co_30_Pt_70_ film into Hall bar devices for electrical transport measurements (see “Methods”). We changed the current angle *θ*_I_ relative to the [1-10] crystal axis to investigate the crystal symmetry-dependent properties, as illustrated in Fig. 2a. *θ*_I_ = 0, 60, 120, 180° and *θ*_I_ = 30, 90, 150° represent that the current is applied along the low-symmetry axes and high-symmetry axes, respectively. Figure 2b shows the anomalous Hall loop of the 6 nm Co_30_Pt_70_ device, which agrees with its good PMA. The current-induced field-free magnetization switching loops for varying *θ*_I_ are shown in Fig. 2c. We found that there is no obvious switching loop when the current is applied along the high-symmetry axes. In contrast, the switching loops appeared when the current is applied along the low-symmetry axes. In addition, the switching loop polarities for two adjacent low-symmetry axes (for example 0 and 60°) are opposite. The switched resistance Δ*R*_I_ is defined to be the difference between the Hall resistance when the current pulse is swept from positive maximum to 0 mA and that when the current pulse is swept from negative maximum to 0 mA. Figure 2d shows the *θ*_I_ dependence of the switching ratio Δ*R*_I_/Δ*R*_H_ of the 6 nm sample, where Δ*R*_H_ is the Hall resistance change in the AHE loop. The 120° periodic switching behavior is consistent with the three-fold in-plane rotational symmetry of Co platelet/Pt at the interface. This three-fold angular-dependence of the field-free magnetization switching also exists in Co_30_Pt_70_ devices with varied thicknesses from 8 to 12 nm (Fig. 2e–g), whose anomalous Hall loops and current-induced magnetization switching loops for different *θ*_I_ values are shown in Supplementary Fig. S4 and S5, respectively. We found that Δ*R*_I_/Δ*R*_H_ decreases with the increasing thickness (Supplementary Fig. S6), which could be explained if the Co platelets mainly exist near the substrate.

**Fig. 3:**
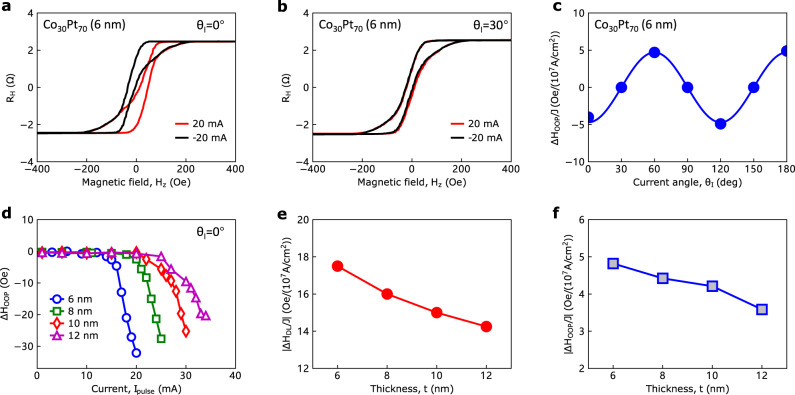
Current-induced out-of-plane and in-plane SOT effective fields. **a**, **b** AHE loops under +20 and −20 mA pulsed currents for *θ*_I_ = 0° (**a**) and 30° (**b**). **c** Current angle dependence of the out-of-plane effective field efficiency (Δ*H*_OOP_/*J*) in 6 nm Co_30_Pt_70._ **d** Current dependence of Δ*H*_OOP_ for various Co_30_Pt_70_ thicknesses. **e**, **f** Thickness dependences of in-plane and out-of-plane SOT effective fields for *θ*_I_ = 0°, respectively. The Δ*H*_OOP_ for the largest measured current in (**d**) is used for the calculation in (**f**).

To reveal the origin of the current-driven switching, we first characterized the out-of-plane effective field (Δ*H*_OOP_) of the devices by measuring the anomalous Hall loops under positive and negative pulsed *d.c*. currents for different *θ*_I_, as shown in Fig. 3a, b and Supplementary Figs. S7. Figure 3c shows the *θ*_I_ dependent out-of-plane effective field efficiency (Δ*H*_OOP_/*J*), by using the Δ*H*_OOP_ measured at 20 mA. The three-fold angular-dependent property is in accordance with that for the current-induced field-free switching. The current dependences of Δ*H*_OOP_/*J* for different Co_30_Pt_70_ thicknesses are shown in Fig. 3d, where we observed different current thresholds above which the Δ*H*_OOP_/*J* values start to emerge and grow rapidly (Supplementary Section 4). We also estimated the in-plane damping-like effective field by harmonic Hall measurements (see Supplementary Sections 5 and 6). Figure 3e and f show that both the in-plane damping-like effective field efficiency (Δ*H*_DL_/*J*) and Δ*H*_OOP_/*J* decrease slowly with increasing thickness. Then we measured the Δ*H*_DL_ and the DMI field (*H*_DMI_) for varying *θ*_*I*_ in our Co_30_Pt_70_ single layer (see Supplementary Section 7). We found that both *H*_DMI_ and Δ*H*_DL_ are isotropic with *θ*_*I*_ (Supplementary Fig. S17), in contrast to the anisotropic behavior of Δ*H*_OOP_. Next, we studied the reversal mode^30^ of the magnetization during the SOT switching. We performed microscopy imaging of the magneto-optical Kerr effect (MOKE) on the device, as shown in Supplementary Section 8. By comparing the experiments with and without external magnetic field, we found that the reversal mode of the field-free switching in our Co_30_Pt_70_ single layer is dominated by domain nucleation. We note that the behavior and the origin of the field-free switching in Co_30_Pt_70_ are quite different from that recently reported in the CoTb layer^25^. First, the field-free switching in ref. ^25^ starts with the nucleation of a domain at the edge of the sample due to the gradient DMI. While the field-free switching of Co_30_Pt_70_ starts with the nucleation of the domains mostly at the center part of the bar device, where the current density is the largest. Second, the antisymmetric DMI-induced field-free switching should have a one-fold angular dependence (with a period of 360°) on the in-plane current angle (*θ*_I_). In contrast, the field-free SOT switching in Co_30_Pt_70_ shows a three-fold angular dependence on *θ*_I_. Therefore, the crystal symmetry, instead of the DMI, plays a key role in the three-fold field-free switching in Co_30_Pt_70_. Furthermore, the *M-H* curves under different in-plane magnetic field directions (Supplementary Fig. S3) do not show any noticeable anisotropy, which rules out the effect of the magnetic easy axis tilting on the free field switching. To study if the change of the substrate can influence the switching behavior or not, we prepared a 6 nm Co_30_Pt_70_ sample on SrTiO_3_ (111) substrate. We found that the switching behavior (Supplementary Fig. S22c) for Co_30_Pt_70_/SrTiO_3_ (111) is almost the same as that (Fig. 2c) for Co_30_Pt_70_/MgO (111), which excludes the influence of substrate change and suggests a self-switching characteristic in the Co_30_Pt_70_ layer.

### Composition dependence of the switching performance

**Fig. 4:**
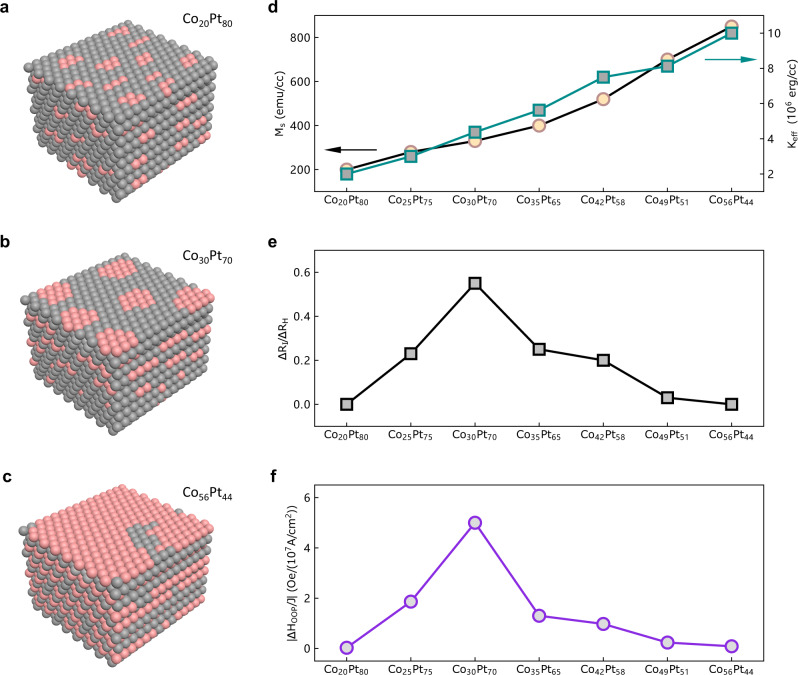
Composition dependence of the OOP effective fields and switching behavior in Co_*x*_Pt_100-*x*_ single layers on SrTiO_3_ (111) substrates. **a**–**c** Tentative structures of Co_20_Pt_80_, Co_30_Pt_70_, and Co_56_Pt_44_, which correspond to the lowest, optimal and highest Co compositions in our experiments, respectively. A large disordered background was omitted in these structures for easier analysis. Since the Co platelets are mainly distributed near the substrate, the top side of the structure in (**b**) is adjacent to the substrate. **d** Composition dependence of *M*_s_ and *K*_eff_ in Co_x_Pt_100-x_ single layers. **e** Composition dependence of Δ*R*_I_/ Δ*R*_H_ in Co_x_Pt_100-x_ single layers. **f** Composition dependence of Δ*H*_OOP_/*J* in Co_x_Pt_100-x_ single layers.

For further study, we investigated the field-free switching behavior in 6 nm Co_*x*_Pt_100-*x*_ on SrTiO_3_ (111) with *x* ranging from 20 to 56. The magnetic and structural properties for these Co_*x*_Pt_1-*x*_ samples are shown in Supplementary Fig. S20 and S21, all showing good PMA and good epitaxial growth. Their XRD results are better than that of Co_30_Pt_70_/MgO (111). In Fig. 4d, the saturation magnetization *M*_s_ and the effective perpendicular anisotropy energy *K*_eff_ increase with increasing Co composition from 20% to 56%, consistent with previous reports^31–33^. We performed the current-induced magnetization switching and OOP effective field measurements for these Co_*x*_Pt_100-*x*_ samples, as shown in Supplementary Fig. S22 and S24. We found that Co_30_Pt_70_ shows the most prominent switching behavior with the highest Δ*R*_I_/Δ*R*_H_ ratio, as summarized in Fig. 4e. With *x* decreasing or increasing from 30, the field-free switching ratio decreases. Figure 4f shows that the largest Δ*H*_OOP_/*J* also appears in Co_30_Pt_70_, following the behavior of Δ*R*_I_/Δ*R*_H_. To interpret the above phenomena, we tentatively make a comparison among three typical compositions (Co_20_Pt_80_, Co_30_Pt_70_, and Co_56_Pt_44_), whose structures are schematically shown in Fig. 4a–c, respectively. For Co_20_Pt_80_, we assume that the Co clusters are small and randomly distributed in the Pt matrix, as shown in Fig. 4a. For Co_56_Pt_44_ in Fig. 4c, alternative stacking of Co and Pt in long-range order can be formed, giving rise to an *L*1_1_ ordered CoPt under certain growth conditions. We note that the nominal chemical ordering parameter (*S*) for the presented Co_56_Pt_44_ structure in Fig. 4c is close to 1. However, in experiments, the *S* value is usually lower than 0.3 because the *L*1_1_ CoPt is a metastable phase. Therefore, a large portion of the structure should be occupied by a randomly distributed Co and Pt (almost fully disordered) background. Similar disordered backgrounds may also exist in Co_20_Pt_80_ and Co_30_Pt_70_. However, since a fully disordered background will not allow for the OOP SOT by symmetry, we ignore its existence in Fig. 4a–c for simplicity. Then we can make the symmetry analysis of the three typical structures. For Co_20_Pt_80_, the small composition ratio of Co makes it difficult to form large Co platelets, and the OOP SOT is unlikely to generate in those randomly distributed small Co clusters. For Co_56_Pt_44_, the long-range atomically ordered [Co/Pt]_N_ is close to *L*1_1_ CoPt, which adopts the point group $R\bar{3}m$ in the bulk. It is only at the substrate/film interface that the point group can reduce to 3m1, which gives rise to a finite 3m torque^19^. In addition, the large saturation magnetization and anisotropy energy in Co_56_Pt_44_ (Fig. 4d) also make it more difficult to be switched compared with the other compositions. For Co_30_Pt_70_, the Co platelets are not enough to form a long-range atomically ordered [Co/Pt]_N_ structure, but can create a relatively large portion of Co platelet/Pt structures. More importantly, the Co platelets mainly distribute within several nanometers near the substrate. It is likely that the numbers of the Co platelets show a decreasing trend from the bottom (near the substrate) to the top (near the surface). This could be the reason why we can have a sizable 3m torque in our Co_30_Pt_70_ sample.

### Origin of the damping-like spin-orbit torque

Next, we explored the origin of in-plane damping-like torque in the Co_30_Pt_70_ single layer. Previously, the *L*1_0_ FePt single layer has been reported to exhibit current-induced bulk SOT due to the composition gradient along the thickness direction^22,23^. The bulk SOT has also been reported to exist in CoTb^24,34^, GdFeCo^35^, disordered-CoPt^36^, and disordered-Fe_*x*_Pt_1-*x*_^37^ single layers. We checked the composition distributions of Co and Pt in the 12 and 6 nm Co_30_Pt_70_ film along the film thickness direction by using high-angle annular dark-field scanning transmission electron microscopy (HADDF-STEM), as shown in Supplementary Fig. S25. We found that the Co/Pt ratio for the 12 nm film changes from 30:70 at the bottom of the film to 27:73 at the top and the composition gradient is estimated to be 0.5%/nm by a linear fitting. The composition gradient of the 6 nm Co_30_Pt_70_ is 1.85%/nm, which is larger than that of the 12 nm Co_30_Pt_70_. This could be one of the reasons why the damping-like effective field of 6 nm is larger than that in 12 nm Co_30_Pt_70_ (Fig. 3e).

To further verify if the composition gradient in the Co_30_Pt_70_ single layer plays the main role in generating the damping-like effective field or not, we fabricated two control samples with designed positive and negative composition gradients along the film’s normal direction. Then we conducted the current-induced switching experiments and found that the switching polarity reverses when the composition gradient is reversed (Supplementary Fig. S26). Because the current-induced magnetization switching under assistant in-plane magnetic field is mainly driven by the damping-like SOT, the reversal of the switching polarity indicates a sign change in the damping-like SOT. Therefore, we concluded that the damping-like SOT in Co_30_Pt_70_ should mainly come from the composition gradient.

## Discussion

If we compare the switching loops of Co_30_Pt_70_ (in Fig. 2c) and that of CuPt/CoPt in our previous work^19^, we found their switching polarities are opposite. For example, when *θ*_I_ = 0°, the switching polarity is clockwise for Co_30_Pt_70_ while anti-clockwise for CuPt/CoPt. Based on our 3m torque model^19^, the sign of the 3m torque (along +*z* or −*z*) is determined by the sign of the damping-like torque. Therefore, the opposite switching polarities for the two systems mean that their damping-like torques have opposite signs. This could be due to the fact that the CuPt spin source layer locates at the bottom of CoPt for the CuPt/CoPt bilayer, while for the Co_30_Pt_70_ single layer, there is Pt segregation on the top side of the layer.

In order to test the robustness of the field-free switching in Co_30_Pt_70_, we performed a switching cycling test by applying positive and negative pulsed currents repeatedly. After 15,000 circles, we observed that Δ*R*_I_ almost keeps unchanged (Supplementary Fig. S27), which indicates a good endurance. We note that the switching ratio for our Co_30_Pt_70_ doesn’t exceed 60%, which could be mainly due to the pinning effect from the magnetic Hall arms. Since it is not possible to make a pillar structure^19^ in the single-layer form, we suggest one can use non-magnetic Hall voltage arms (such as Ti/Cu electrodes) to avoid the pinning effect and improve the switching ratio^38^.

In summary, we report the observation of the current-induced field-free switching in Co_*x*_Pt_100-*x*_ single layers. By studying the SOT in Co_*x*_Pt_100-*x*_ with varying *x* from 20 to 56, we found that the Co_30_Pt_70_ has the largest OOP effective field efficiency and best field-free switching performance. The origin of the field-free switching in Co_30_Pt_70_ is attributed to the formation of Co platelets and the composition gradient in the film’s normal direction. The composition gradient along film normal direction gives rise to the in-plane damping-like torque while the low symmetry (3m1) property at the interface of Co platelet/Pt gives rise to the 3m torque. The cooperation of these two effects leads to a three-fold field-free switching in the Co_30_Pt_70_ single layer. Our result of the self-switching in Co_30_Pt_70_ has provided one of the most simplified structures for field-free switching of perpendicular magnetization. The good endurance and high thermal stability make it a good candidate for magnetic memory devices and other spintronic applications. Our work may stimulate further investigation of the current-induced self-switching in single-layer systems.

## Methods

### Sample growth and device fabrication

Co_*x*_Pt_100-*x*_ single layers were epitaxially deposited on MgO (111) and SrTiO_3_ (111) single-crystal substrate by *d.c*. magnetron sputtering (AJA). The base pressures were lower than 4 × 10^−8^ Torr. The Ar gas pressure was kept a constant at 5 mTorr. The temperature was kept at 300 °C during the deposition process, then the film was left to cool down to room temperature and a 2 nm SiO_2_ protection layer was deposited as protection. After deposition, the films were patterned into 5 μm Hall bars with different in-plane orientations by using laser writer and Ar ion milling. Then the contact electrode pattern was defined by laser writer, followed by the deposition of Ti (7 nm)/Cu (100 nm) and the lift-off process.

### Current-induced switching measurement

For the current-induced magnetization switching measurement, a pulsed *d.c*. electrical current with a duration of 30 μs was applied. After 8 s, the Hall resistance was recorded by using a small *a.c*. excitation current (50 μA).

### Current-induced out-of-plane effective field measurement

For each data point in the AHE loop when measuring the out-of-plane effective field, we first swept the external out-of-plane magnetic field to a target value (such as *H*_z_ = 20 Oe), then we applied a current pulse (such as *I*_pulse_ = 18 mA) with a pulse width of 30 μs. After 6 s of the applied pulsed current, we applied a small *ac* current to detect the Hall resistance.

### TEM sample preparation and characterization

TEM samples of the Co_30_Pt_70_ single layer were fabricated by a focused ion beam machine (FEI Versa 3D system). The sample was thinned down using a Ga ion beam first with an accelerating voltage of 30 kV and then 8 kV. After that, the sample was polished by a 2 kV ion beam. Microstructures were studied by transmission electron microscopy (TEM) (JEOL JEM-2100F) at an accelerating voltage of 200 kV with energy-dispersive X-ray spectroscopy (EDS) analysis performed in scanning transmission electron microscopy (STEM) mode.

## Supplementary information

Supplementary Information

## Data Availability

The data that support the findings of this study are available from the corresponding author upon reasonable request.
