# Supplementary material for: Current-induced self-switching of perpendicular magnetization in CoPt single layer
Source: Nat Commun. 2022 Jun 20;13:3539. doi: 10.1038/s41467-022-31167-w (PMC9209536; doi:10.1038/s41467-022-31167-w)
Supplement: Supplementary file 1 — Supplementary Information [file 41467_2022_31167_MOESM1_ESM.pdf]

Supplementary materials

for

Current-induced self-switching of perpendicular magnetization in CoPt single layer

Liang Liu[1†], Chenghang Zhou[1†], Tieyang Zhao[1], Bingqing Yao[2], Jing Zhou[1], Xinyu Shu[1], Shaohai Chen[1], Shu Shi[1], Shibo Xi[3], Da Lan[1], Weinan Lin[1], Qidong Xie[1], Lizhu Ren[4], Zhaoyang Luo[1], Chao Sun[1], Ping Yang[3,1], Er-Jia Guo[5], Zhili Dong[2], Aurelien Manchon[6], Jingsheng Chen[1,7,8,9*]

[1]*Department of Materials Science and Engineering, National University of Singapore, Singapore 117575*

[2]*School of Materials Science and Engineering, Nanyang Technological University, Singapore 639798*

[3]*Singapore Synchrotron Light Source (SSLS), National University of Singapore, 5 Research Link, Singapore 117603*

[4]*Department of Electrical and Computing Engineering, National University of Singapore, Singapore 117583*

[5]*Beijing National Laboratory for Condensed Matter Physics and Institute of Physics, Chinese Academy of Sciences, Beijing 100190, China*

[6]*Aix-Marseille Univ, CNRS, CINaM, Marseille, France*

[7]*Suzhou Research Institute, National University of Singapore, Suzhou 215123, China*

[8] *Chongqing Research Institute, National University of Singapore, Chongqing 401120, China*

[9]*Institute of Material Research and Engineering,A*STAR,Singapore 138634, Singapore*

[†]These authors contributed equally to this work.

*email: msecj@nus.edu.sg

1. Structural properties of the $Co_{30}Pt_{70}$ on MgO (111) substrate.

2. Magnetic properties of the $Co_{30}Pt_{70}$ single layer.

3. Angular dependence of the current-induced magnetization switching in the $Co_{30}Pt_{70}$ single layers.

4. Out-of-plane effective field in the $Co_{30}Pt_{70}$ single layer

5. In-plane effective field in the $Co_{30}Pt_{70}$ single layer.

6. Thermal electrical effects

7. Angular dependences of the DMI field and the current-induced effective fields in the $Co_{30}Pt_{70}$ single layer

8. Magneto-optical Kerr effect (MOKE) image of the $Co_{30}Pt_{70}$ single layer

9. Composition dependence of the current-induced magnetization switching in Co_xPt_{100-x}

10. Composition gradient direction and switching polarity in $Co_{30}Pt_{70}$ single layer

11. Endurance test in $Co_{30}Pt_{70}$ single layer

1. Structural properties of the $Co_{30}Pt_{70}$ on MgO (111) substrate.

In our experiments, the samples have a stack of MgO/$Co_{30}Pt_{70}$ (t_{CoPt})/ SiO_2 from bottom to top and MgO is the substrate. In general, the formula of Co-Pt can have several structures: A1-(Coz, Pt), $L1_0$-CoPt, $L1_1$-CoPt, $L1_2$-$CoPt_3$, A3-(Co,Pt), B_h-CoPt, $D0_{19}$-Co_3Pt. To verify the structure of our CoPt, we checked the stacking sequence by HR-TEM (Fig. 1b). It is known that an *fcc* structure has an ABCABC… atomic stacking sequence of the close-packed plane along the perpendicular direction, whereas that for an *hcp*-based crystal is ABAB…. According to the TEM image in Fig. 1b, we observed an ABCABC… stacking sequence, which is consistent with an *fcc*-CoPt crystal structure. The three-fold symmetry from $Co_{30}Pt_{70}$ (002) plane rotating along the [111] direction was observed in the patterns of Fig. 1d, which further confirms the *fcc* structure $Co_{30}Pt_{70}$.

Figure S1a,b display the X-ray absorption near edge spectra (XANES) which reflects the local structure around Co and Pt. The extracted structure parameters are presented in Table S1. In general, The Co-Co bonds should not exist in an ideally ordered $L1_2$ structure, and the number is randomly 3 in fully disordered $Co_{30}Pt_{70}$. Here, the Co-Co pairs (N) is increased in in-plane compared to that in out-of-plane, which indicates the formation of Co platelets.

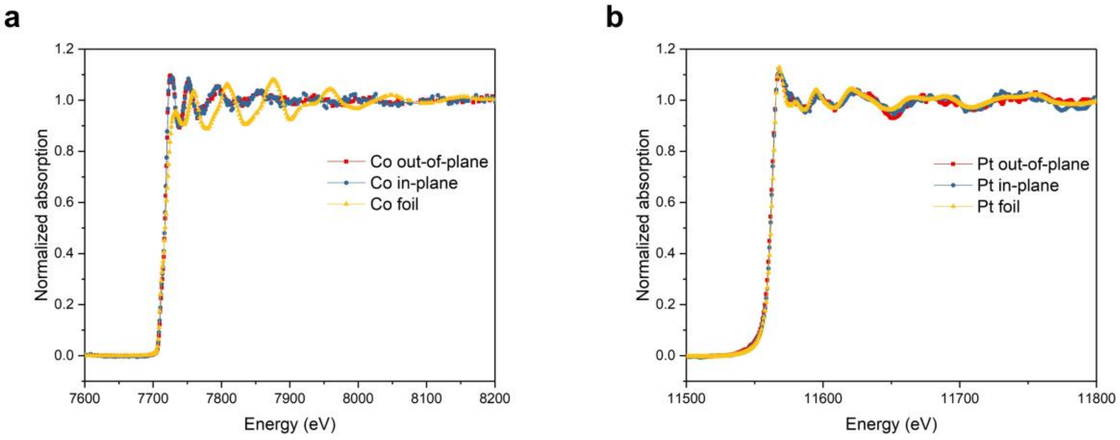

Figure S1| XANES of $Co_{30}Pt_{70}$ thin film. **a**, Co K-edge XANES for Co foil and for the $Co_{30}Pt_{70}$ film. **b,** The Corresponding Pt L3 edge XANES for Pt foil and the film.

Table S1. Structures of 12 nm $Co_{30}Pt_{70}$ with XAFS fitted data extracted from Fig. S1

	Bond	R(Å)	N
		XAFS	
in-plane	Co-Pt	2.687	6.57
	Co-Co	2.734	1.91
out-of plane	Co-Pt	2.684	7.00
	Co-Co	2.610	1.28
in-plane	Pt-Pt	2.774	5.57
	Pt-Co	2.687	2.19
out-of-plane	Pt-Pt	2.765	9.70
	Pt-Co	2.684	2.33

2. Magnetic properties of the $Co_{30}Pt_{70}$ single layer.

Figure S2 shows the magnetic hysteresis loops of the un-patterned $Co_{30}Pt_{70}$ films with thicknesses ranging from 6 to 12 nm. The square out-of-plane hysteresis loops and small in-plane hysteresis loops indicate their good perpendicular magnetic anisotropy. The saturation magnetization of the $Co_{30}Pt_{70}$ is about 330 emu/cc. The effective perpendicular anisotropy energy in decreases from 5.1 to 1.7 erg/cc, as summarized in Fig. 1f.

We measured the in-plane *M-H* loops of an un-patterned 6 nm $Co_{30}Pt_{70}$ film, with the magnetic field applied along different in-plane directions (θ_H=0, 30, 60, and 90 deg) with respect to the [1-10] axis, as shown in Fig. S3. We found neither obvious in-plane effective field nor anisotropic behavior, which rules out the effects of the magnetic easy axis tilting on the free field switching. Fig. S4 shows the Anomalous Hall Effect of $Co_{30}Pt_{70}$ with varied thicknesses. Fig. S4a shows that the coercive fields and the Hall resistances of the 6 nm $Co_{30}Pt_{70}$ with different θ_I from 0 deg to 180 deg remain almost unchanged, which indicates the good uniformity of the devices. Similar phenomena were also observed in thicker $Co_{30}Pt_{70}$ (Fig. S4b-d).

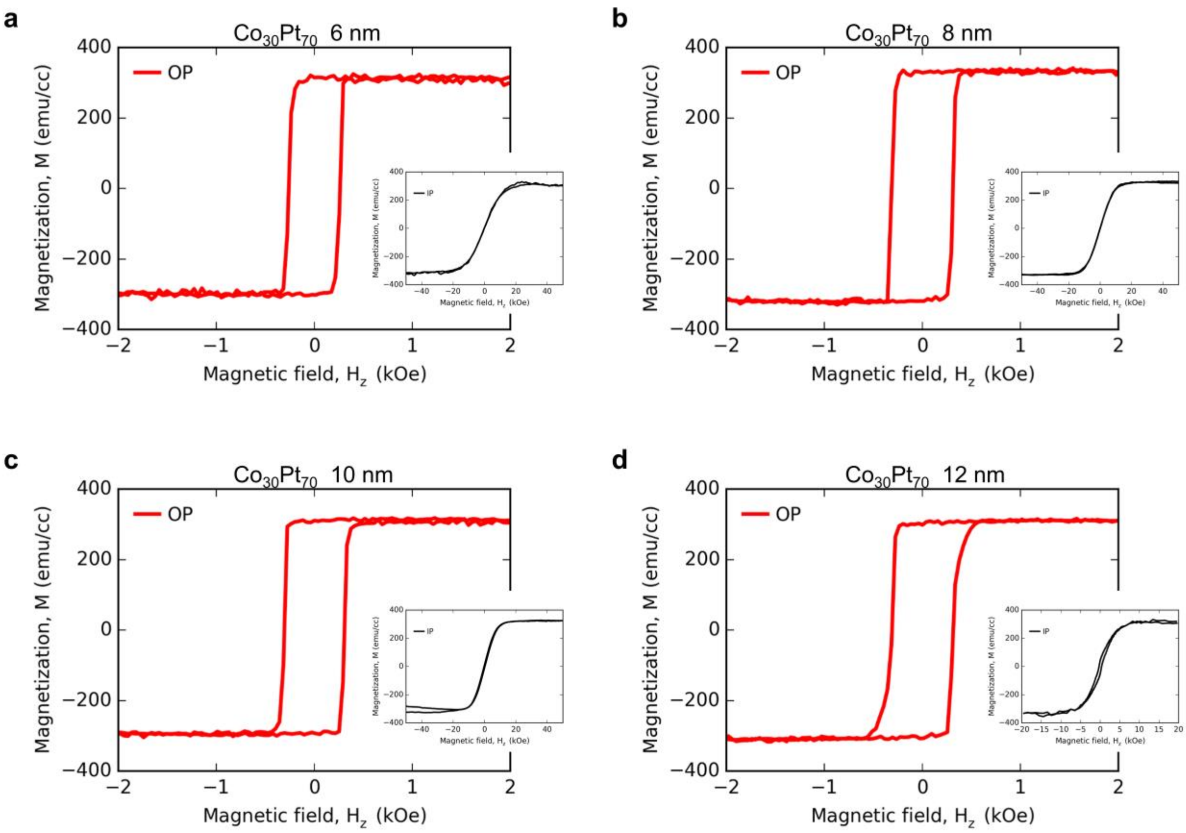

Figure S2| Magnetic properties of the $Co_{30}Pt_{70}$ films on MgO (111) substrate. a-d, Out-of-plane and in-plane magnetic hysteresis loops of un-patterned CoPt thin films with thicknesses from 6 to 12 nm.

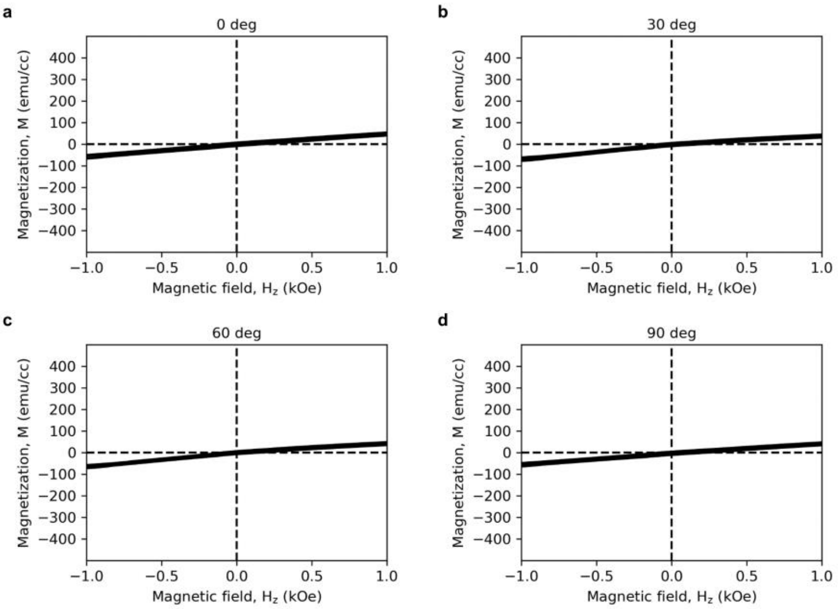

Figure S3| In-plane *M-H* loop of the 6 nm $Co_{30}Pt_{70}$ with different in-plane magnetic field angles.

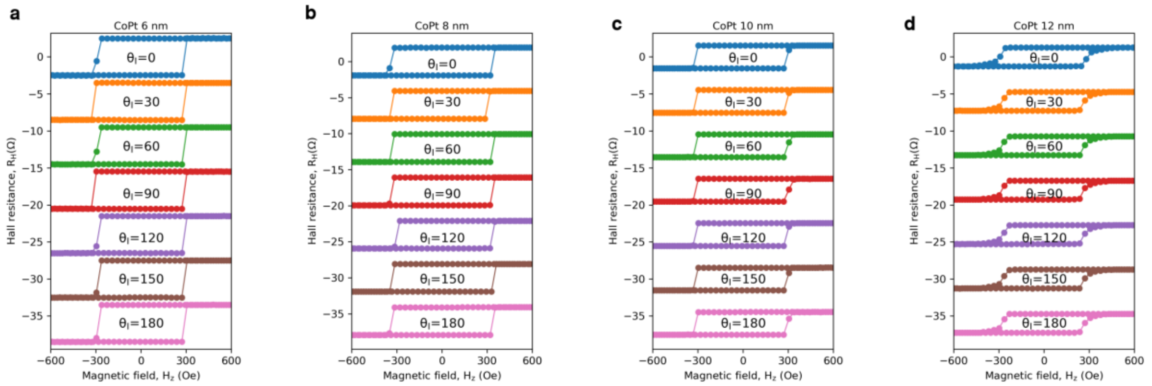

Figure S4| Anomalous Hall loops of $Co_{30}Pt_{70}$ with four thicknesses.

3. Angular dependence of the current-induced magnetization switching in the $Co_{30}Pt_{70}$ single layers.

Figure S5 shows the thickness dependence of the current-induced magnetization switching of the $Co_{30}Pt_{70}$ single layers (8 nm, 10 nm, and 12 nm) for different θ_I ranging from 0 deg to 180 deg. When the pulsed current is along the low-symmetry axes (θ_I =0, 60, 120, and 180 deg), the switched Hall resistance is maximized. In contrast, when the pulsed current is along the high-symmetry axes (θ_I =30, 90, and 150 deg), there is no switching loop. The thickness dependence of the switching ratio is summarized in Fig. S6.

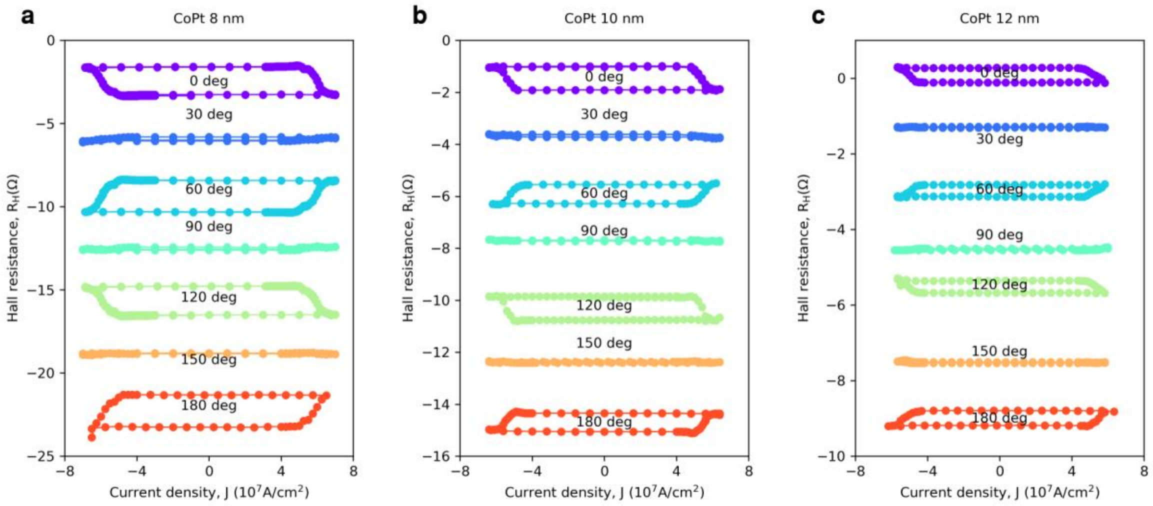

Figure S5| Current-induced magnetization switching for different current angles in 8, 10,

and 12 nm $Co_{30}Pt_{70}$.

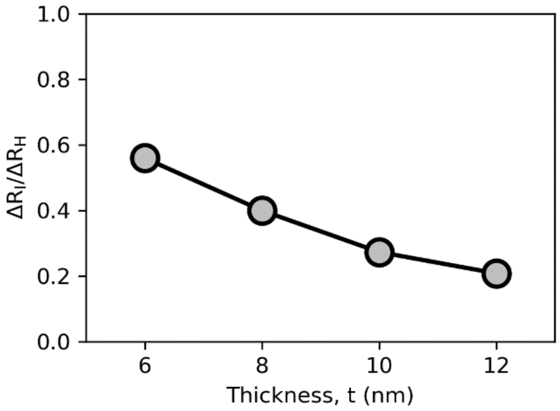

Figure S6| Thickness dependence of $\Delta R_I/\Delta R_H$ for $Co_{30}Pt_{70}$.

4. Out-of-plane effective field in the $Co_{30}Pt_{70}$ single layer

Figure S7 shows the AHE loops under positive and negative currents for different θ_I ranging from 60 deg to 180 deg in $Co_{30}Pt_{70}$ (6 nm). ΔH_{OOP} is estimated from the Hall loop shift. When the current is +20 mA, the shift is positive for $\theta_I = 120$ and negative for $\theta_I = 60$ and 180 deg. For $\theta_I = 30$, 90, and 150 deg, there is no shift.

Figure S8-10 shows the results of the out-of-plane effective field measurement with film thicknesses ranging from 8 to 12 nm. The thickness dependence of $\Delta H_{OOP}/J$ is summarized in Fig. 3f.

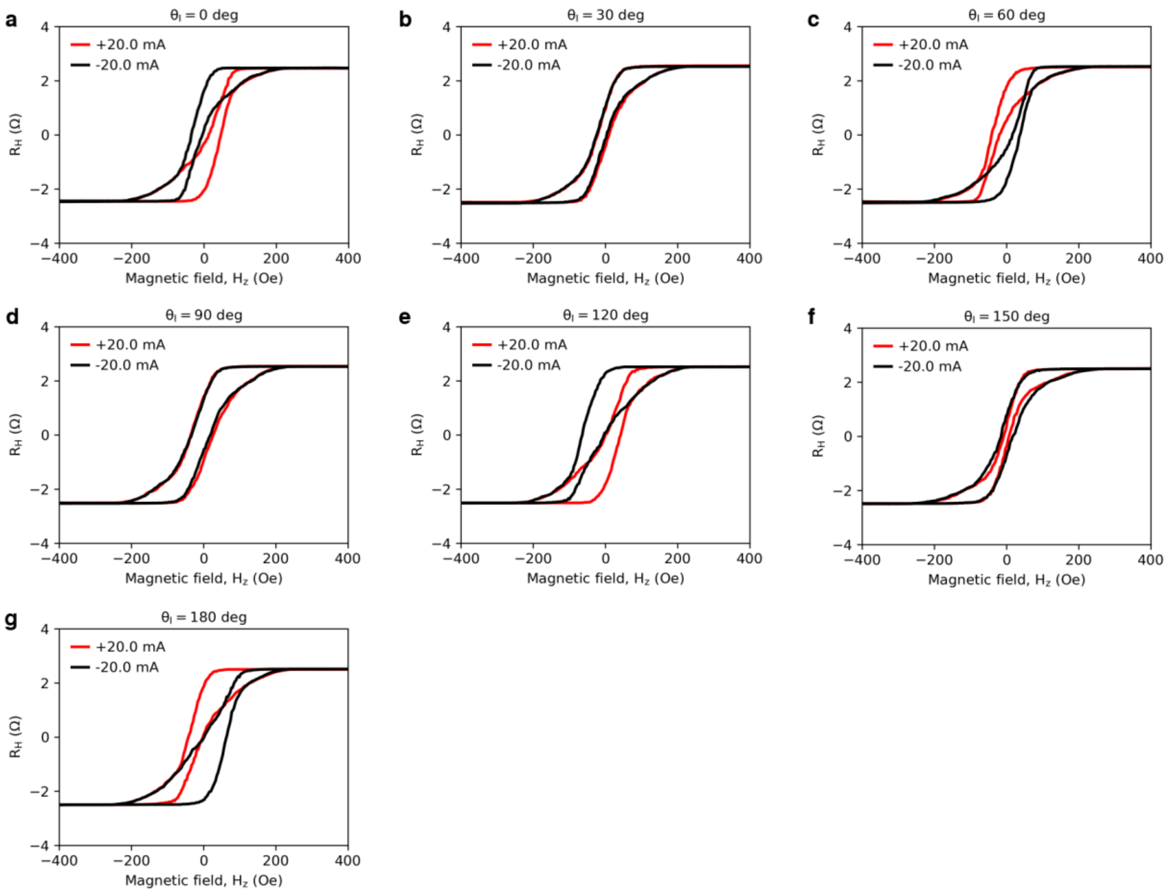

Figure S7| a-e, Anomalous Hall loops under +20 mA and -20 mA pulsed d.c. current for varying θ_I in 6 nm $Co_{30}Pt_{70}$.

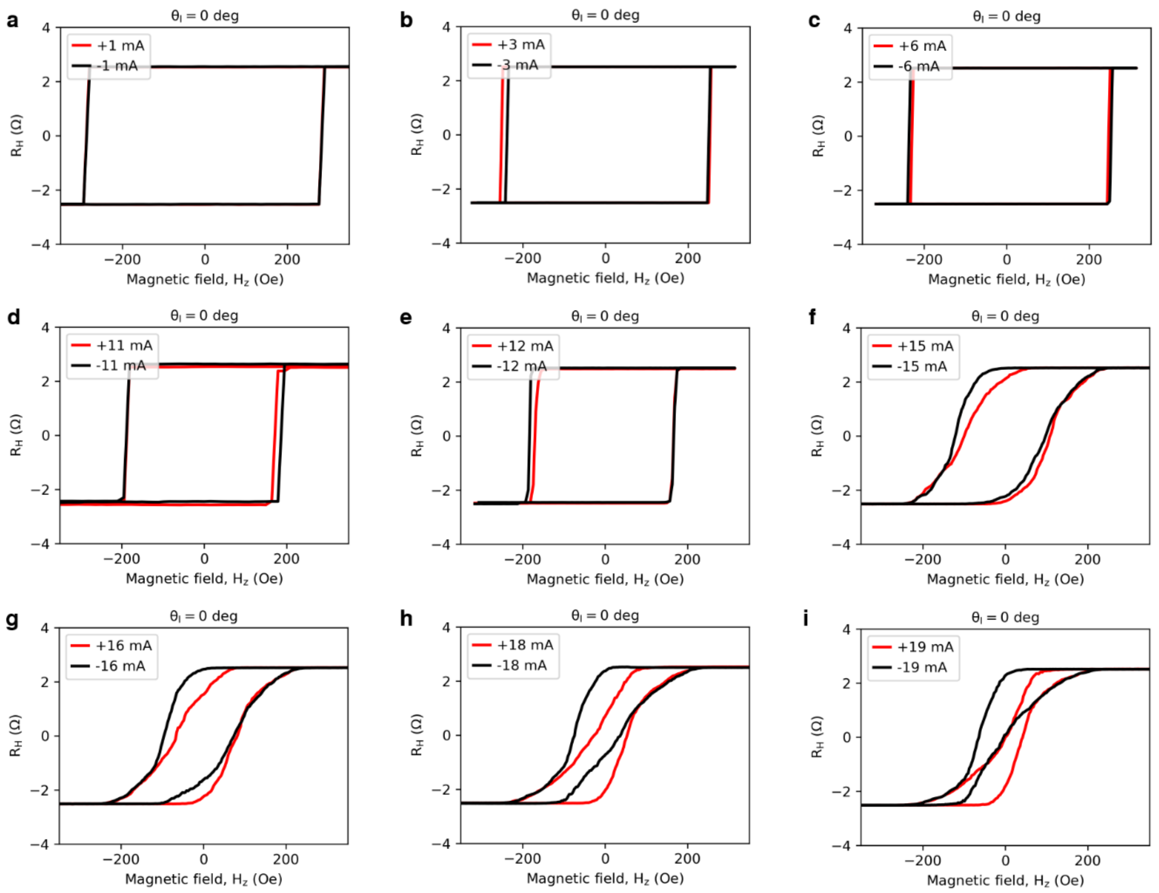

Figure S8| Anomalous Hall loops under different currents in 6 nm $Co_{30}Pt_{70}$ with θ_I = 0 deg.

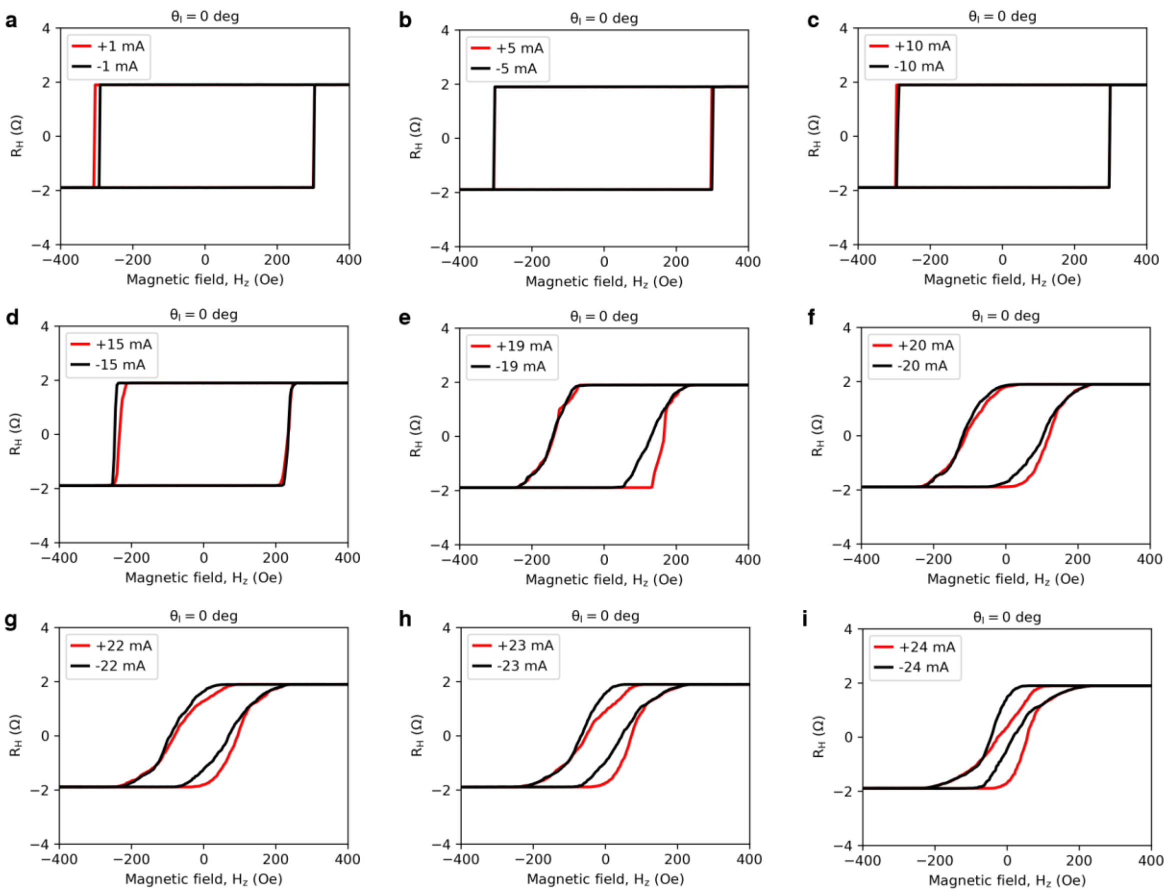

Figure S9| Anomalous Hall loops under different currents in 8 nm $Co_{30}Pt_{70}$ with θ_I = 0 deg.

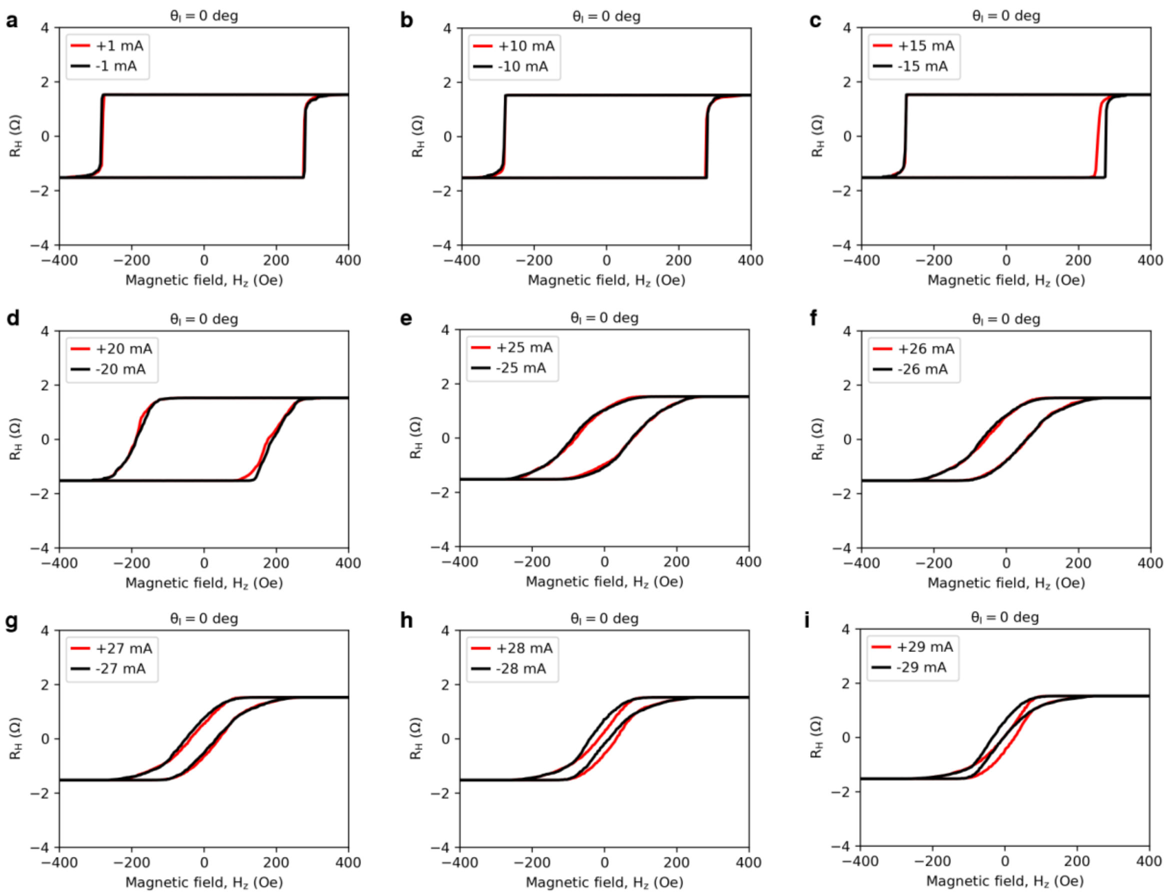

Figure S10| Anomalous Hall loops under different currents in 10 nm $Co_{30}Pt_{70}$ with $\theta_I = 0$ deg.

5. In-plane effective field in the $Co_{30}Pt_{70}$ single layer.

We performed harmonic Hall voltage analysis[1] on the sample. An alternating current (*ac*) of frequency 317.3 Hz is applied to the Hall-bar device to induce small oscillations of magnetization around its equilibrium direction. The in-phase first harmonic signal (V_ω) and out-of-phase second harmonic signals ($V_{2\omega}$) are measured simultaneously using two lock-in amplifiers. The current-induced longitudinal and transverse effective field components are defined as ΔH_L and ΔH_T. After deducing the thermoelectric effects (Supplementary Fig. S12-13). the current-induced effective fields are expressed as

$$\Delta H_{L(T)} = -2\frac{B_{L(T)} \pm 2\delta B_{T(L)}}{1-4\delta^2}$$

Where $B_{L(T)}$ is defined as $\left\{\frac{\partial V_{2\omega}}{\partial H_{ext}} / \frac{\partial^2 V_{\omega}}{\partial H_{ext}^2}\right\}_{H_{ext} \,/\!/\, L(T)}$, δ is the ratio of R_{AHE}/R_{PHE}, which is less than 20 % in our $Co_{30}Pt_{70}$ single layer.

Figure S11 shows the current dependence of the damping-like effective field of the $Co_{30}Pt_{70}$ single layers with thicknesses ranging from 6 nm to 12 nm. We found that ΔH_{DL} decreases with increasing thickness, which is summarized in Fig. 3e.

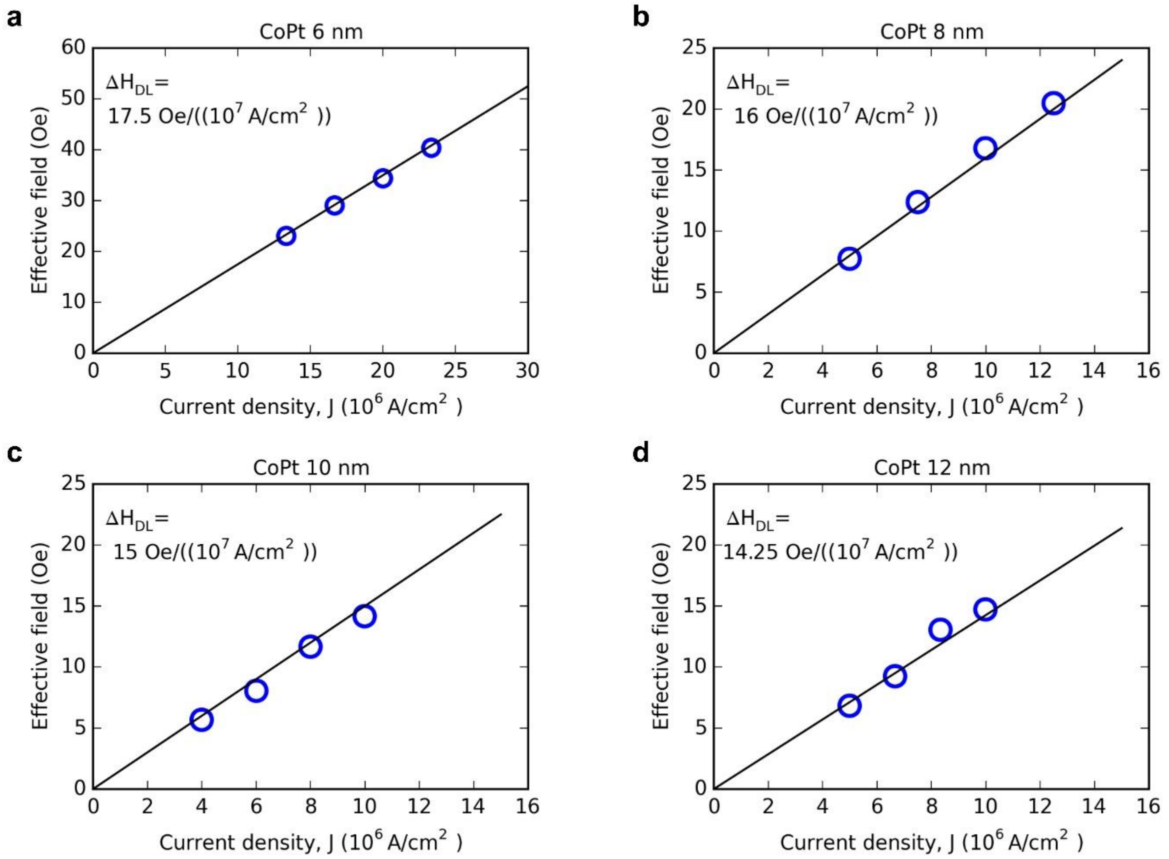

Figure S11| Determination of the in-plane damping-like effective fields in $Co_{30}Pt_{70}$ single layers with varied thicknesses from 6 nm to 12 nm.

6. Thermal electrical effects

The thermoelectric effect and the misalignment of the Hall voltage lead can also contribute to the second harmonic Hall signals. When the current flows through the Hall bar, a temperature gradient $\boldsymbol{\nabla}T = (\nabla T_x, \nabla T_y, \nabla T_z)$ is generated in the $Co_{30}Pt_{70}$ devices. The temperature gradient

generates voltage signals due to anomalous Nernst effect (ANE) and spin Seebeck effect (SSE).

Firstly, we considered the temperature gradients in the film plane. We have measured the second harmonic and first harmonic signals by applying 4 mA a.c. current, as shown in Fig. S8a and Fig. S8b, respectively. Both the anomalous Nernst effect (estimated in Fig. S8a) and the offset contribute to the second harmonic Hall measurement when the magnetic field sweeps in the film plane. However, they can only contribute constant values to the second harmonic signals as shown in Fig. S8c and S8d, which will not affect the result of $\frac{\partial V_{2\omega}}{\partial H}$ in the estimation of ΔH_{DL}.

Secondly, we measured the thermoelectric effect which comes from the perpendicular temperature gradient ∇T_z by sweeping large magnetic field (larger than the anisotropic field H_k) in the film plane. The second harmonic signal can be expressed as:

$$R_{xy}^{2\omega} = \frac{R_{\mathrm{AHE}}}{2}\frac{H_{\mathrm{DL}}}{H_{\mathrm{x}}-H_{\mathrm{k}}} + \frac{R_{\mathrm{PHE}}}{2}\frac{H_{\mathrm{FL}}}{H_{\mathrm{x}}} + R_{\mathrm{ANE+SSE}},$$

where R_{AHE} and R_{PHE} are the anomalous Hall resistance and planar Hall resistance, respectively. Since R_{PHE} (0.7 Ω) is much lower than R_{AHE} (5 Ω) in our $Co_{30}Pt_{70}$ thin film, the second term can be neglected. Then we can extract the signal of SOT and thermoelectric effect by plotting the second harmonic Hall signal as a function of $1/(H_x - H_k)$. The field dependence of first and second harmonic Hall signals with different a.c. currents (1 mA, 2 mA, 4 mA, 6 mA) are shown in Fig. S9a and Fig. S9b. Fig. S9c shows the plot of second harmonic Hall signals as a function of $1/(H_x$-$H_k)$, where we can obtain a linear relationship and the intercept can be interpreted as $R_{\mathrm{ANE+SSE}}$. Since $V_{\mathrm{ANE+SSE}} \sim \nabla T_z \times M_x$ in longitudinal measurement, so we have $V_{\mathrm{ANE+SSE}} \sim M\sin\theta_M$, where θ_M is the polar angle of the magnetization. Then we calculated the contribution of $V_{\mathrm{ANE+SSE}}$ in the small field range, as shown in Fig. S8d. The change of signal is 80 nV with fixed 4 mA a.c. current, which is less than 5 % of the overall second harmonic signal (1.6 μV) in Fig. S8c. We confirmed that the thermoelectric effect contribute a small correction to the results of SOT effective field.

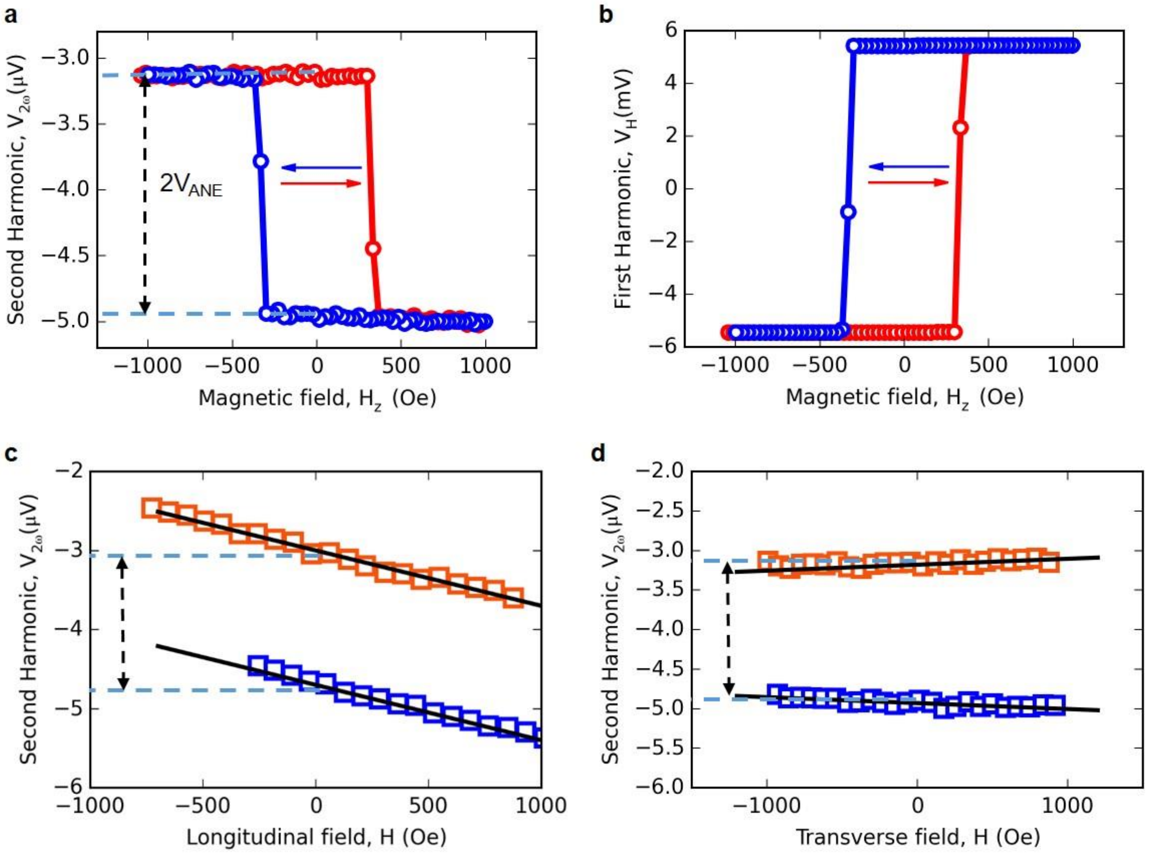

Figure S12| Measurement and subtraction of anomalous Nernst effect in $Co_{30}Pt_{70}$ single layer with θ_I = 0 deg. a, b, Second and first harmonic signals by sweeping out-of-plane magnetic field. **c, d,** Second harmonics signals by sweeping the magnetic field along the longitudinal and transverse direction, respectively.

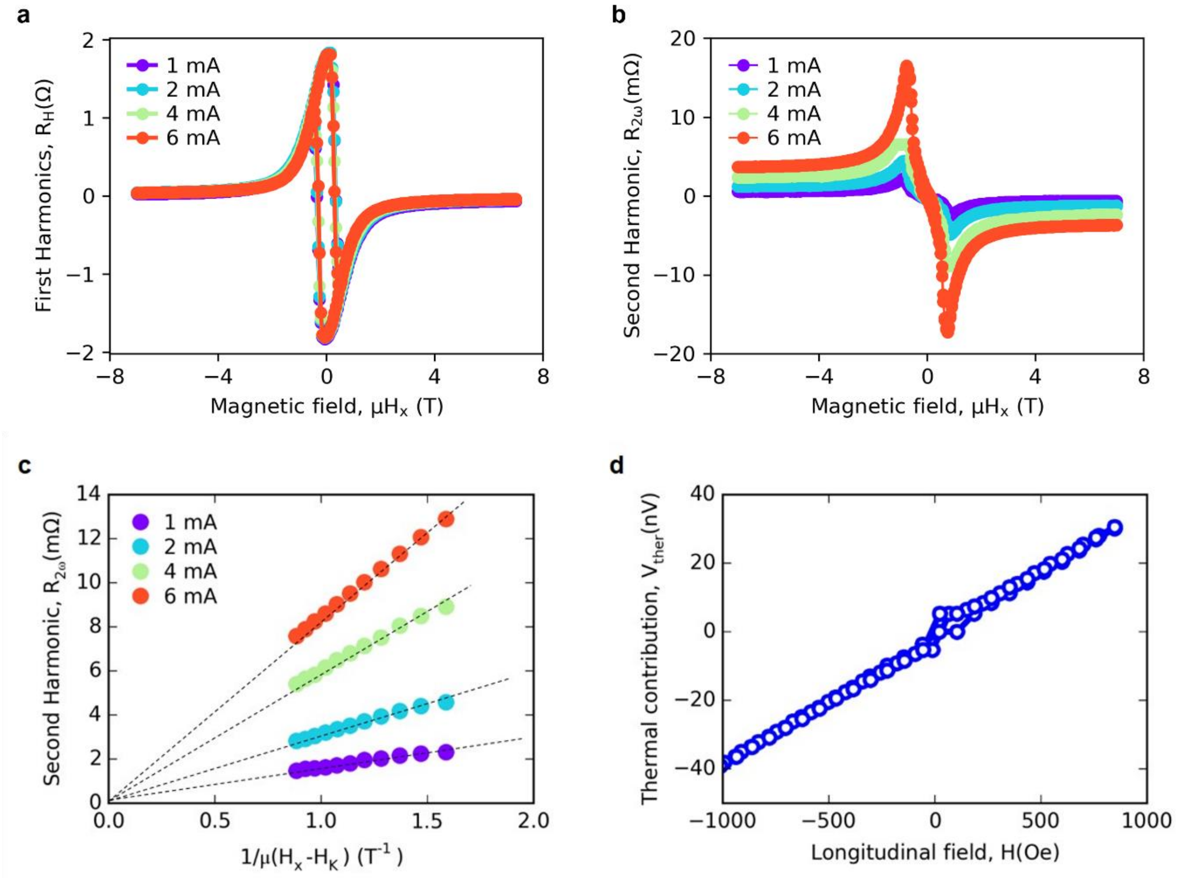

Figure S13| Estimation of the thermal electrical effect from large field fitting of second harmonic Hall signals in $Co_{30}Pt_{70}$ single layer with θ_I = 0 deg. a, b, First and second harmonic signals as a function of the longitudinal magnetic field for different a.c. currents, respectively. **c,** Second harmonic signal as a function of $1/\mu(H_x - H_K)$, and the dashed lines are fits to the data. **d,** Current dependence of the thermal electrical signal.

7. Angular dependences of the DMI field and the current-induced effective fields in the $Co_{30}Pt_{70}$ single layer

To obtain the DMI field, we measured the anomalous Hall loops with positive and negative dc electrical currents under varying in-plane magnetic fields (H_x). Fig. S14 shows the result for a 10 nm $Co_{30}Pt_{70}$ with Co/Pt ratio decreasing from bottom to up along film normal direction. The loop shift indicates an out-of-plane effective field ΔH_{OOP}, which was found to firstly

increase and then saturate with the magnitude of H_x, as summarized in Fig. S16a. The DMI field can be estimated by the H_x value where ΔH_{OOP} starts to saturate[2,3], which means all moment directions within the domain wall are aligned with the external magnetic field. We have also measured the AHE loop of $Co_{30}Pt_{70}$ single layer with Co/Pt ratio increasing along film normal direction (from bottom to up), as shown in Fig. S15. The opposite shift of the AHE loops compared with Fig. S14 indicates that the DMI field is reversed under the reversal of the composition gradient. We have also measured the DMI field for other current angles (θ_I=30, 60, 90 deg) in Fig. S16b-d. We found that the results (both ΔH_{OOP} and the DMI field) are almost independent on θ_I.

In Fig. S17a-c, we summarized the DMI field (H_{DMI}), the in-plane effective field (H_{DL}), and the out-of-plane effective field (ΔH_{OOP}) as a function of θ_I. We found that both H_{DMI} and H_{DL} are isotropic with θ_I. In contrast, ΔH_{OOP} shows an anisotropic behavior, or more specifically, a three-fold angular dependence. Therefore, in our case, the gradient-induced DMI is not responsible for the three-fold field-free switching.

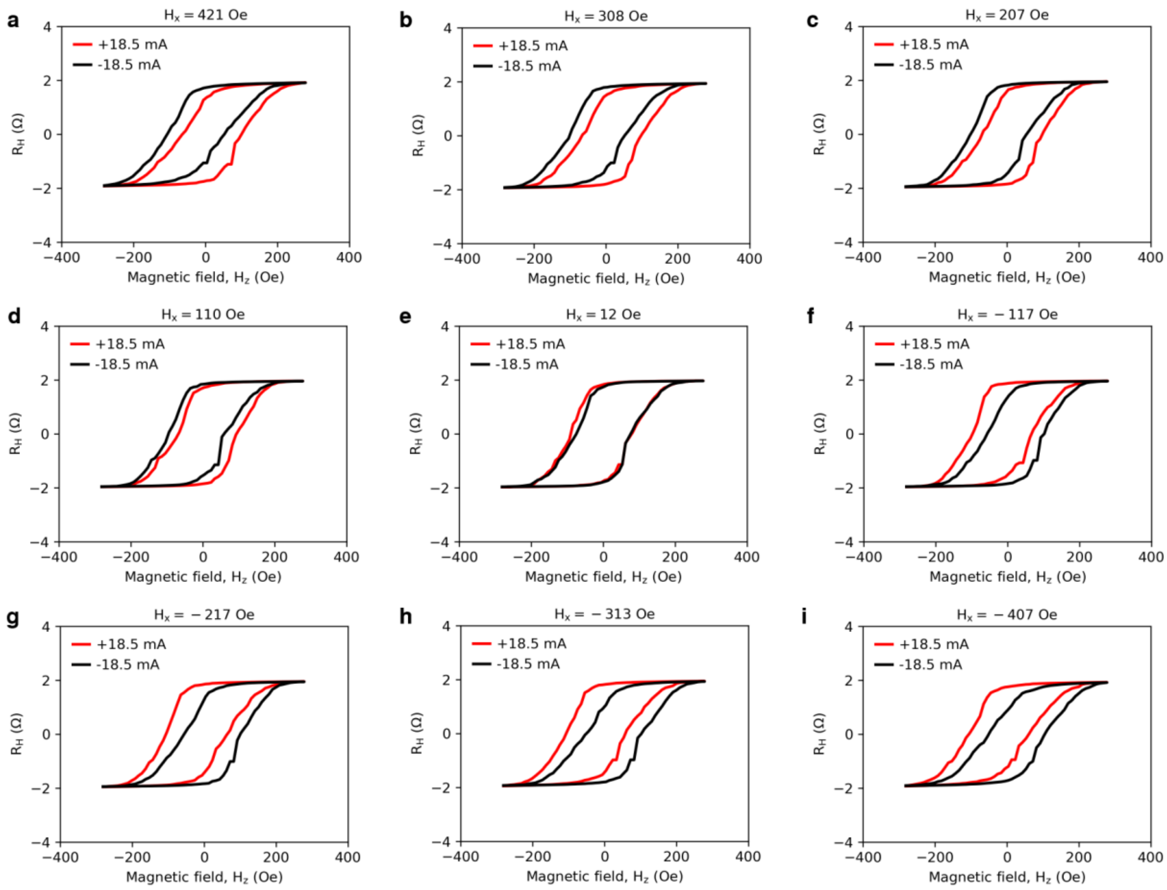

Figure S14| AHE loops for +18.5 mA and -18.5 mA currents under different in-plane magnetic fields in 10 nm $Co_{30}Pt_{70}$ single layer with a decreasing Co/Pt ratio from bottom to top along film normal direction. The current angle θ_I=0.

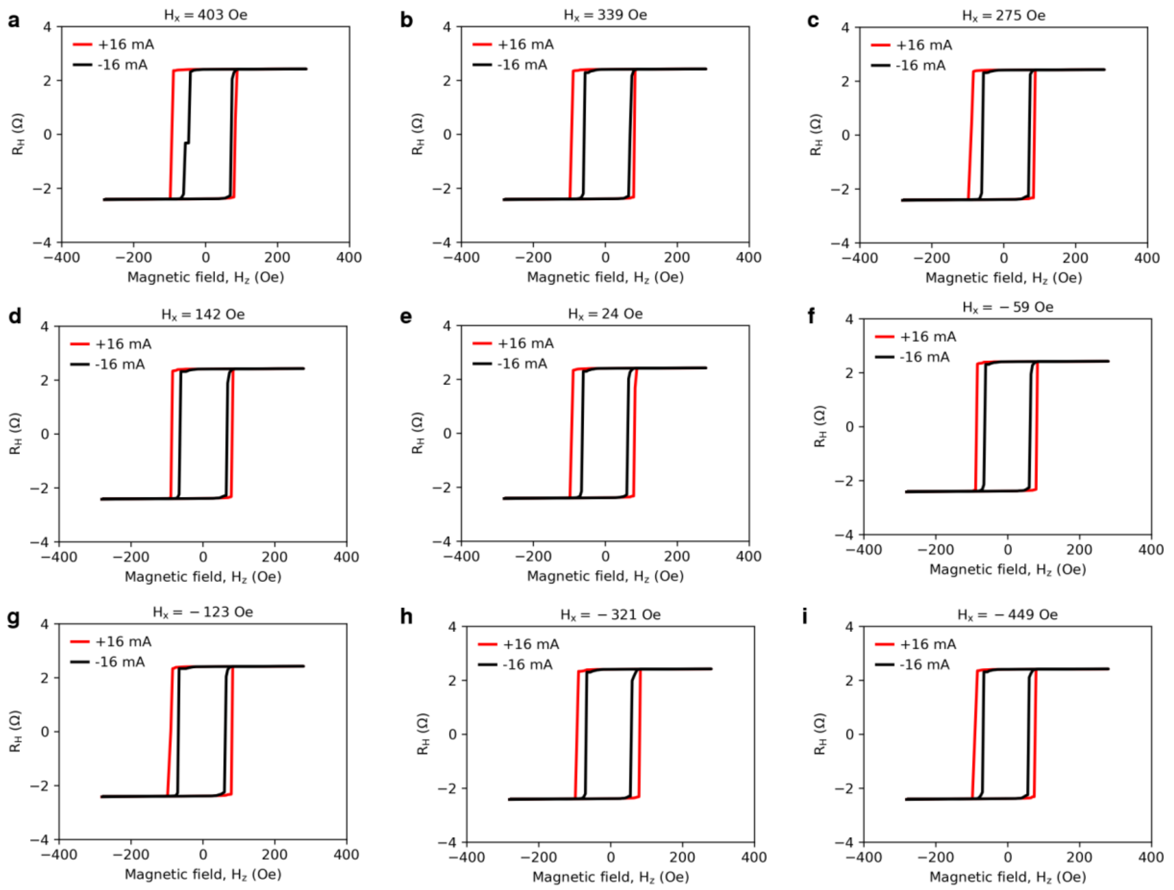

Figure S15| AHE loops for +16 mA and -16 mA currents under different in-plane magnetic fields in 6 nm $Co_{30}Pt_{70}$ single layer with an increasing Co/Pt ratio from bottom to top along film normal direction. The current angle θ_I=0.

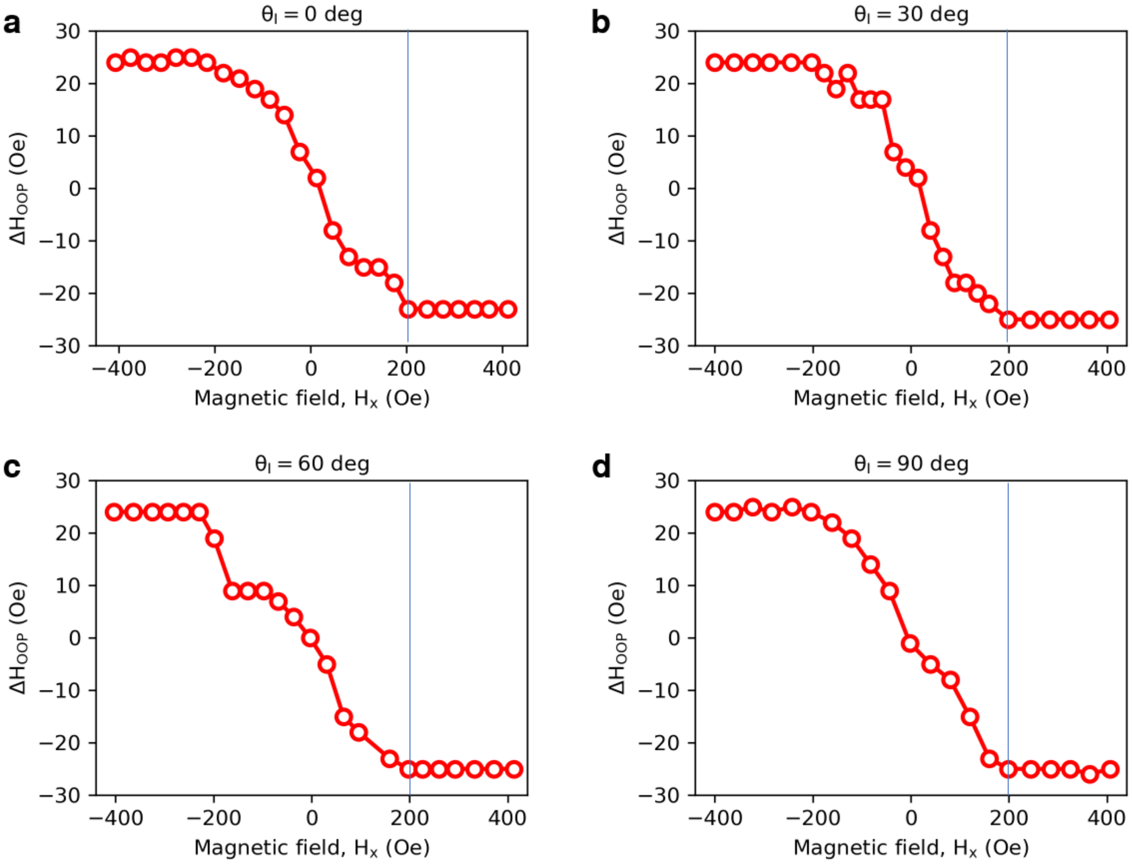

Figure S16| Current-induced out-of-plane effective fields for a 10 nm $Co_{30}Pt_{70}$. a-d, OOP effective field as a function of the in-plane magnetic field for θ_I = 0, 30, 60 and 90 deg, respectively.

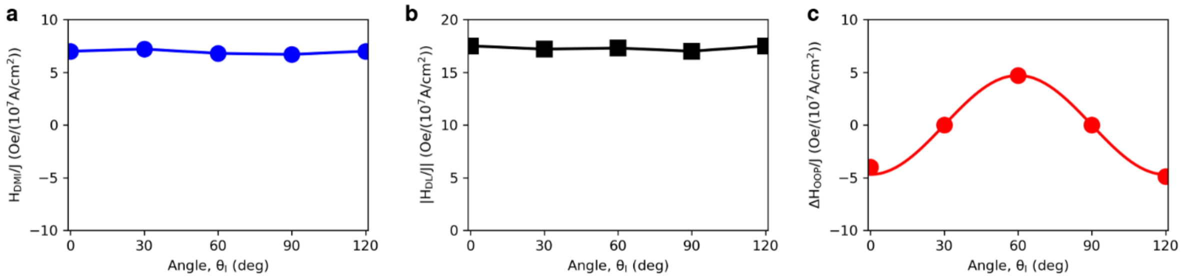

Figure S17| a, Current angle dependence of the DMI field for the 10 nm sample. b,c, Current angle dependences of the in-plane damping like effective field (b) and out-of-plane effective field (c) for the 6 nm sample.

8. Magneto-optical Kerr effect (MOKE) image of the $Co_{30}Pt_{70}$ single layer

Figure S18 and Figure S19 shows the MOKE image of SOT switching in $Co_{30}Pt_{70}$ single layer with and without external magnetic field. For H_x=0 Oe, the time dependence of the magnetization process is shown in Fig. S18. We applied a series of current pulses and the number (N) of the pulse is indicated on the left side of the figure. After the application of each pulse, the MOKE image was captured to check the magnetization distribution on the device. When $0<I_{pulse}<14.03$ mA, we could not observe any signal change. When I_{pulse}=14.03 mA, we found that there exists a nucleated domain after the application of one pulse (N=1). As N increased to 30, the domain number was kept unchanged. With I_{pulse} increased to 14.06 mA, we found that there are two isolated domains for N=1. As N increased to 3, five isolated domains were observed. Then with N increasing from 4 to 30, the MOKE image almost didn't change. The process from N=0 to N=3 showed a typical magnetization reversal mode dominated by nucleation[4]. Then we increased I_{pulse} to 14.3 mA and 14.5 mA, we found the first pulse (N=1) generated several isolated domains, and many of them merged, resulting in the expansion of the domains.

For H_x=200 Oe, the critical current for magnetization reversal decreases to 13.5 mA. We found the first pulse (1*30 μs) generates an reversed domain on the right side. Then with N increased from 1 to 30, the domain area kept moving towards the left side, which shows a typical mode of domain wall motion. For I_{pulse}=14.2 mA and 14.5 mA, similar domain wall types were observed.

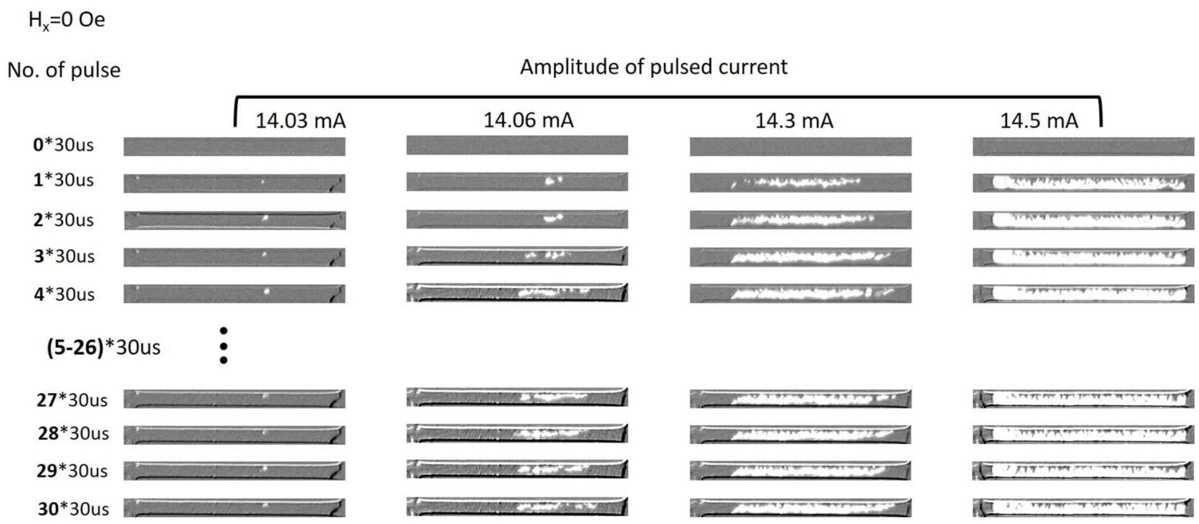

Figure S18| MOKE imaging of the magnetic structure of CoPt single layer during SOT switching without external field (H_x =0 Oe)

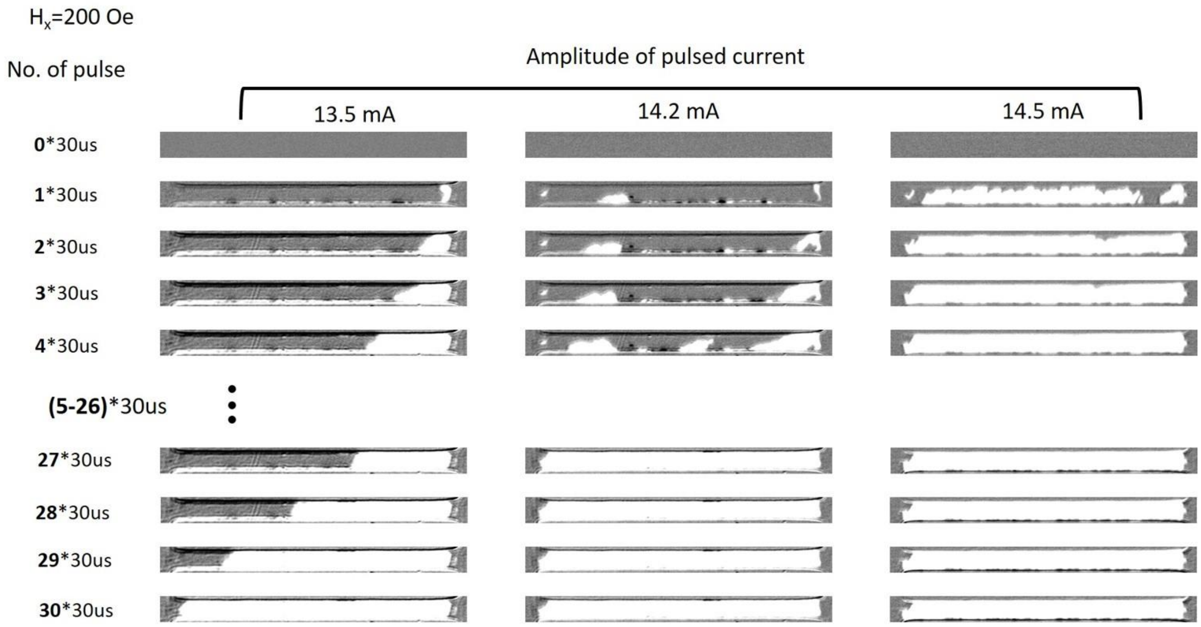

Figure S19| MOKE imaging of the magnetic structure of CoPt single layer during SOT switching without external field (H_x =200 Oe)

9. Composition dependence of the current-induced magnetization switching in Co_xPt_{100-x}

We investigated the composition-dependent switching behavior of Co_xPt_{100-x} single layers. We first measured the magnetic hysteresis loops of un-patterned Co_xPt_{100-x} films. The square

out-of-plane and small in-plane loops indicate the perpendicular magnetic anisotropy of Co_xPt_{100-x} with x ranging from 20 to 56, which is shown in Fig. S20. Fig. S21a shows the HR-XRD pattern of Co_xPt_{100-x} with CoPt (111) peak shift to the right when x increases from 20 to 56. The phi scan of $Co_{56}Pt_{44}$ still shows a 3-fold rotation symmetry (Fig. S21b). Fig. S22 shows the current-induced field-free switching of Co_xPt_{100-x} with varied Co composition. We found that the switching behavior is most prominent for $Co_{30}Pt_{70}$. With Co composition increasing or decreasing the switching behavior become weaker. For $Co_{20}Pt_{80}$ and $Co_{56}Pt_{44}$, the switching tends to vanish. In Fig. S22b-f, the three-fold angular dependence of the switching was observed.

We measured the in-plane damping-like effective field with varied Co compositions, as shown in Fig. S23. We found that ΔH_{DL} fluctuated at around 15 Oe/(A/cm^2) within a certain range, which behaves differently from the composition-dependent SOT switching (characterized by $\Delta R_I / \Delta R_H$) in Fig. 4e.

We also measured the OOP field for different Co compositions, as shown in Fig. S24 and summarized in Fig. 4f of the main text. We found that the OOP field and the switching ratio show a similar trend with Co composition.

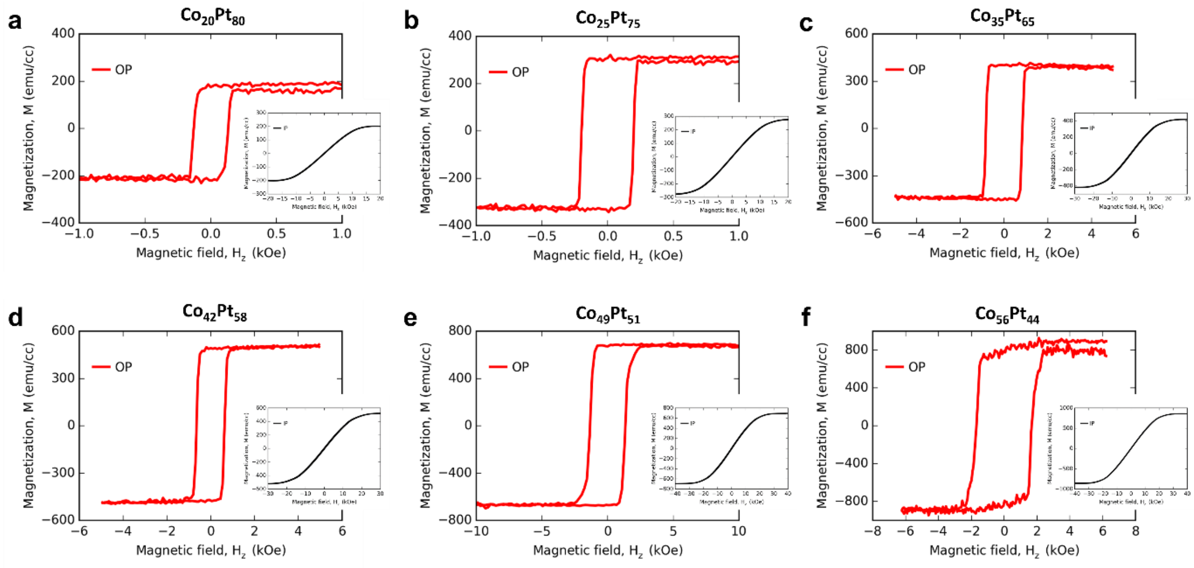

Figure S20| Magnetic hysteresis loops of the Co_xPt_{100-x} samples on STO (111) substrate. a-

f, Out-of-plane and in-plane magnetic hysteresis loops of un-patterned 6 nm Co_xPt_{100-x} thin films with x changing from 20 to 56.

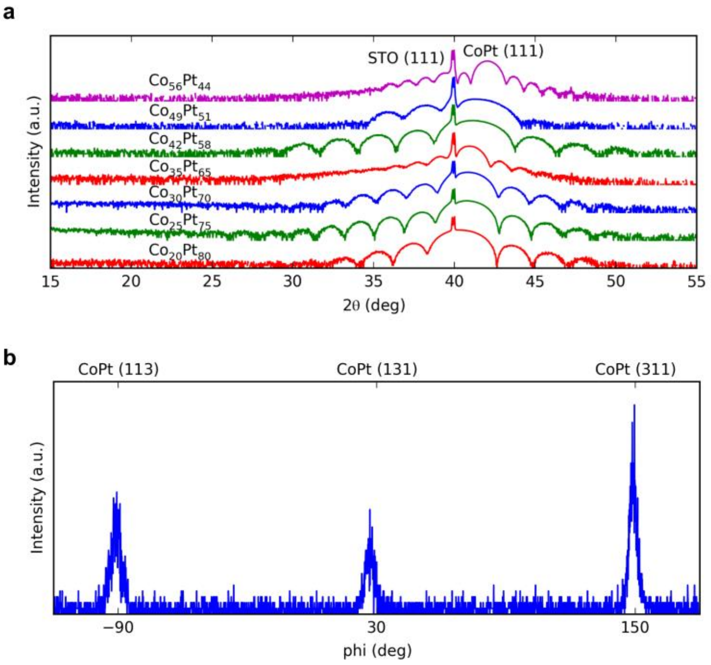

Figure S21| Structure characterization of Co_xPt_{100-x} thin film. a, High-resolution X-ray diffraction (HR-XRD) pattern of different Co_xPt_{100-x} samples. **b,** HR-XRD phi-scan pattern of $Co_{56}Pt_{44}$ (113) plane rotated along [111] axis.

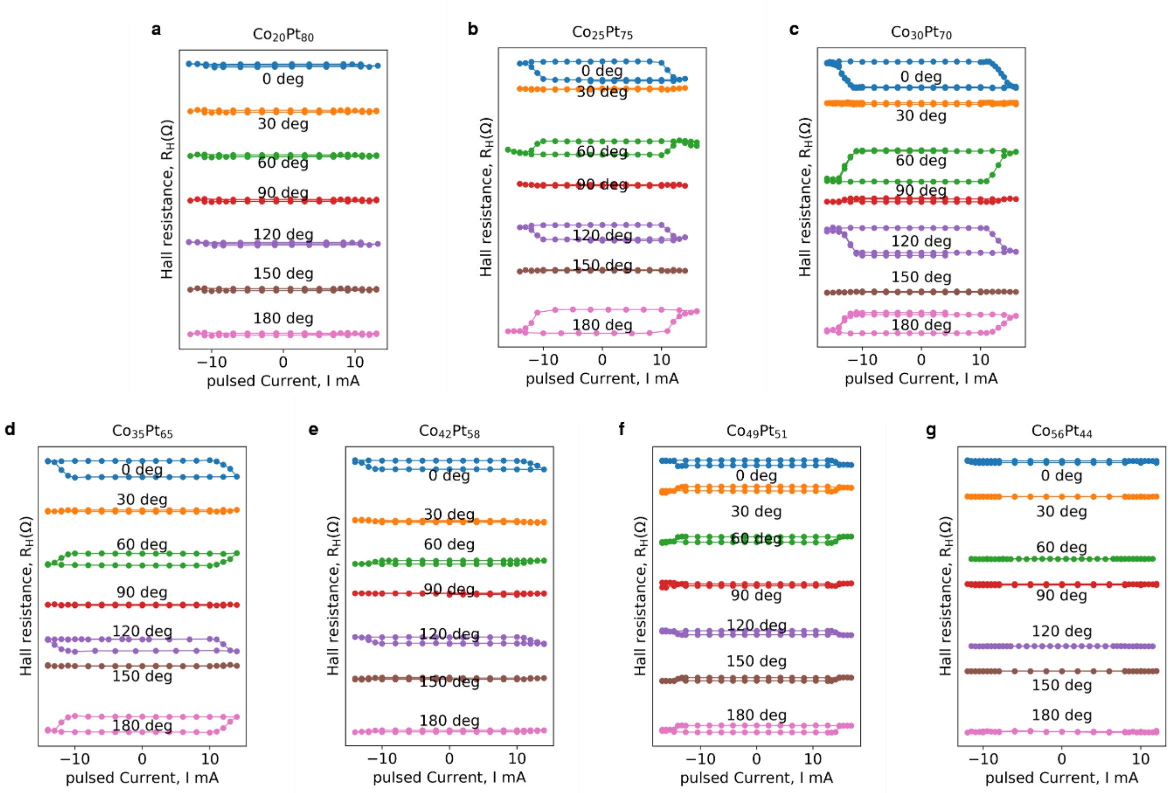

Figure S22| Current-induced field-free magnetization switching in Co_xPt_{100-x} single layers on $SrTiO_3$ (111) substrate, with x changing from 20 to 56.

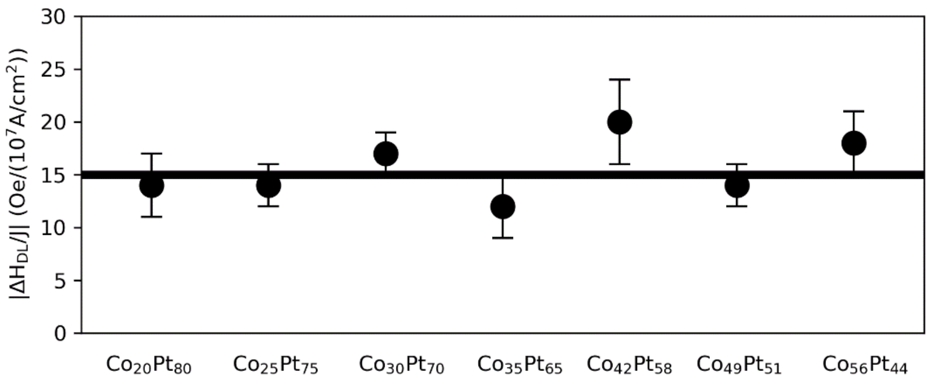

Figure S23| Composition dependence of the in-plane damping like effective field.

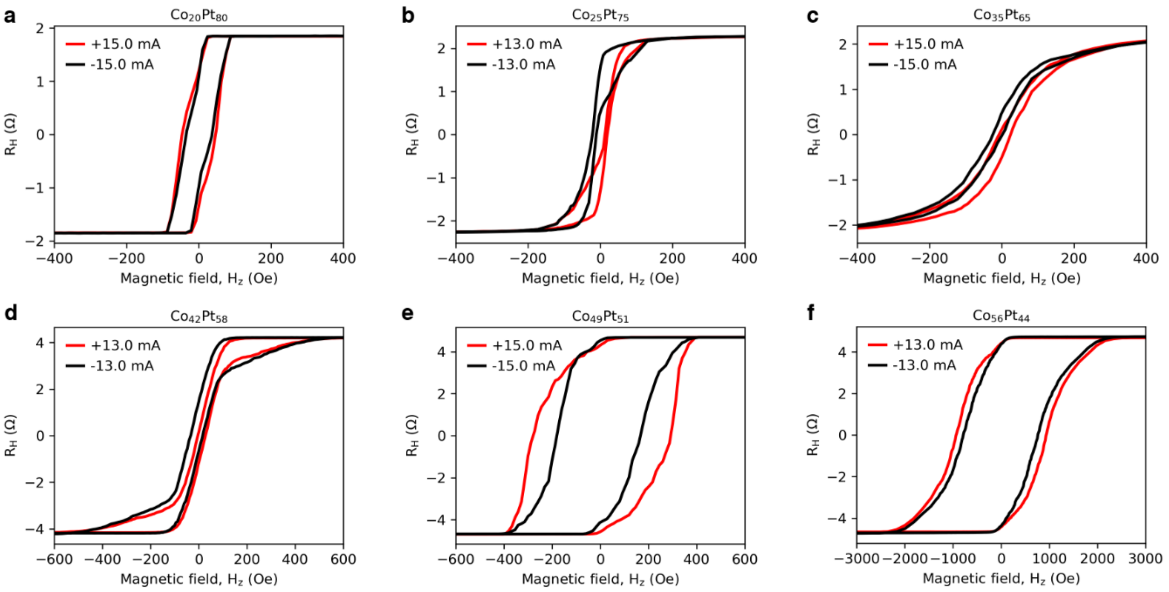

Figure S24| AHE loops under positive and negative currents in Co_xPt_{100-x} single layers. The current is applied along the low-symmetry axis.

10. Composition gradient direction and switching polarity in $Co_{30}Pt_{70}$ single layer

We performed the TEM experiments to check the structure and composition of 6 nm and 12 nm $Co_{30}Pt_{70}$ single layers. The EDX elemental mapping for Co and Pt across the whole film is shown in Fig. S25b and Fig. S26c, respectively. We analyzed the element composition along film normal direction by selected mapping scan, as shown in Fig. S25a. We observed that there is a clear Co:Pt composition gradient along the perpendicular direction with map scan in both 6 nm and 12 nm films, which are shown in Fig. S25d and Fig. S25e, respectively. We define the composition gradient to be the average of the Co/Pt change ratio in 1 nm thickness. We estimated that the composition gradient is around -0.5% per nm in 12nm $Co_{30}Pt_{70}$ film, which is lower than that (-1.85%/nm) in 6 nm $Co_{30}Pt_{70}$ film.

We studied the current-induced magnetization switching in two control samples with purposely controlled signs of composition gradient, as shown in Fig. S26. Fig. S26a shows the structure of the $Co_{30}Pt_{70}$ sample we studied (in Fig. 2 and Fig. 3 of the manuscript), where the Co composition is decreasing from the bottom to the top surface (as evidenced in Fig. S25e in

the supplementary materials). Then we measured the current-induced SOT switching of this $Co_{30}Pt_{70}$ sample under an in-plane magnetic field H_x of 400 Oe or -400 Oe, as shown in Fig. S26d. In experiments, one can purposely control the composition gradient direction. During the thin film deposition, we gradually decreased or increased the sputtering power of Co while fixing the sputtering power of Pt, and the composition ratios were controlled to that shown in Fig. S26b or Fig. S26c, respectively. Therefore, both Fig. S26a and Fig. S26b have a decreasing trend of Co composition along the +z direction, while Fig. S26c has an increasing trend of Co composition along the +z direction. Then we measured the current induced switching for the two control samples in Fig. S26b and Fig. S26c and their results are shown in Fig. S26e and Fig. S26f, respectively. We found that Fig. S26e and Fig. S26d share the same switching polarity. In contrast, the switching polarity in Fig. S26f is opposite to that in Fig. S26e. Therefore, we concluded that the switching polarity is determined by the composition gradient direction.

Because the current-induced magnetization switching under an assistive in-plane magnetic field (±400 Oe) is mainly driven by the damping-like SOT, the switching polarity change indicates a sign change of the damping-like SOT under the sign change of composition gradient direction. Since the substrates for the three samples are the same, we concluded that the damping-like SOT in CoPt should mainly come from the composition gradient instead of the interface effect.

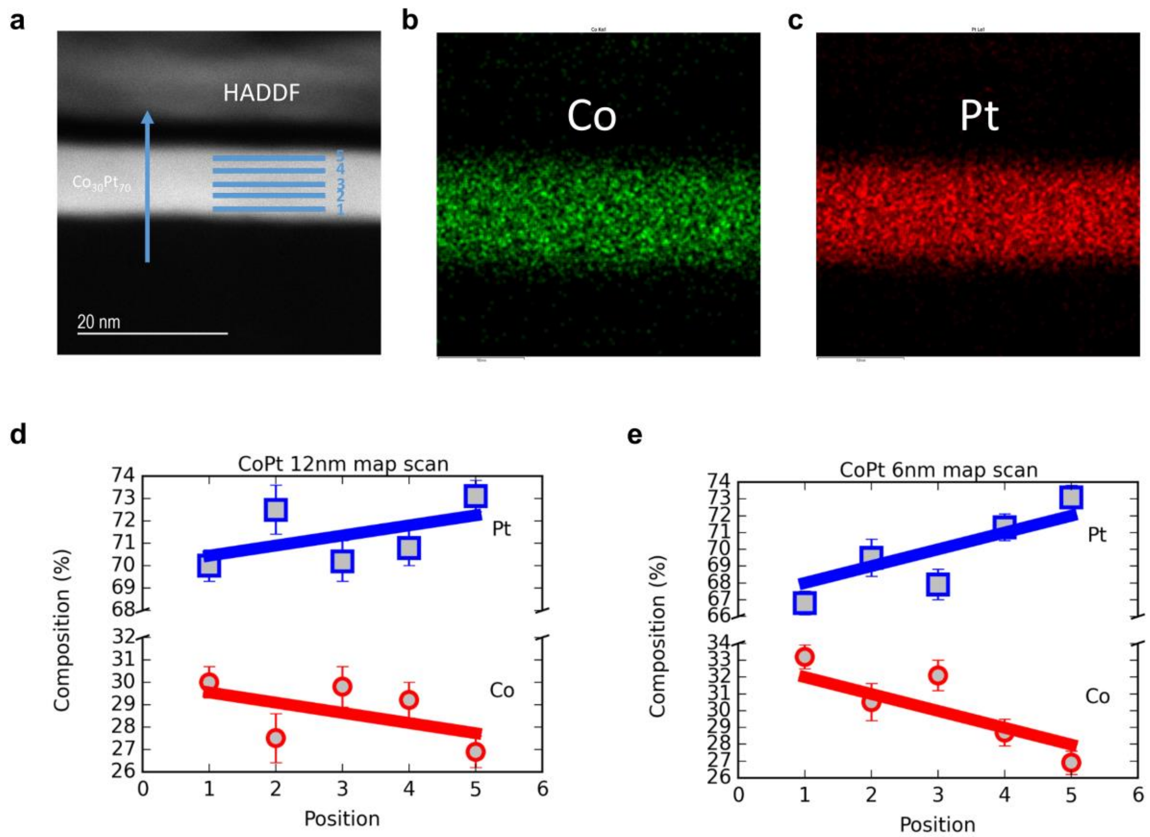

Figure S25| Composition gradient in $Co_{30}Pt_{70}$ single layer. a, Cross-sectional high-angle annular dark-field-STEM image of a 12 nm $Co_{30}Pt_{70}$ film on MgO substrate. **b, c,** Co Kα and Pt Lα edge EDS mappings, respectively. **d, e,** Composition of Co and Pt with the map scan in 12 nm and 6 nm $Co_{30}Pt_{70}$ film, respectively.

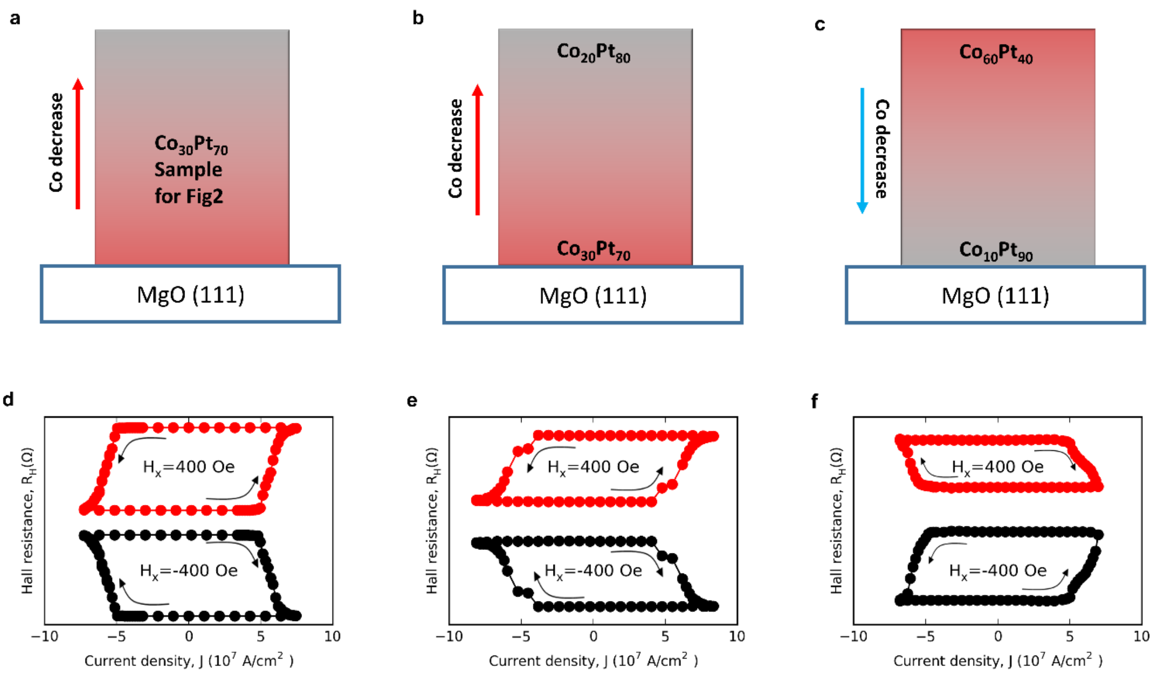

Figure S26| Current induced magnetization switching for CoPt single layers with opposite composition gradient directions. a, $Co_{30}Pt_{70}$ sample with decreasing Co along the +z direction. **b,** Designed CoPt sample with decreasing Co along the +z direction. c**,** Designed CoPt sample with increasing Co along the +z direction. **d-f,** Current-induced magnetization under positive and negative external in-plane magnetic fields for the three samples in **a-c**, respectively.

11. Endurance test in $Co_{30}Pt_{70}$ single layer

We measured the field-free switching cycles in CoPt single layer, and the result is shown in Fig. S27. First, we applied two successive positive pulses and recorded the Hall resistance (R_H) with a small ac current (I_{ac}=50 μA) after a stabilization time of 8 s. Then, we applied two successive negative pulses and measured the stabilized R_H. Therefore, one cycle contains two positive and negative pulses. The high Hall resistance (HR) and low Hall resistance (LR) correspond to the positive and negative current, respectively. After 15000 cycles, the switching behavior remains stable.

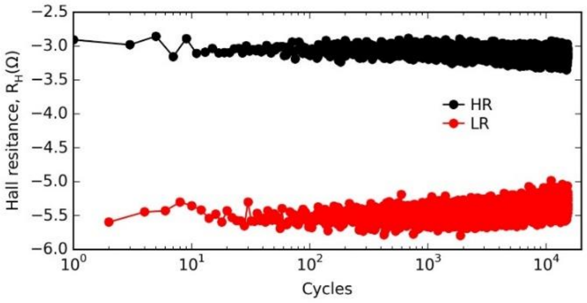

Figure S27| Switching cycling test of $Co_{30}Pt_{70}$.

References

1 Garello K, *et al.* Symmetry and magnitude of spin orbit torques in ferromagnetic heterostructure. *Nat. Nanotechnol.* **8**, 587 (2013).

2 Thiaville, A. *et al.* Dynamics of Dzyaloshinskii domain walls in ultrathin magnetic films. *Europhys. Lett.* **100**, 57002 (2012).

3 Ding, S. *et al.* Interfacial Dzyaloshinskii-Moriya interaction and chiral magnetic textures in a ferrimagnetic insulator. *Phys. Rev. B.* **100**, 100406(R) (2019).

4 Labrune, M. *et al.* Time dependence of the magnetization process of RE-TM alloys. *J. Magn. Magn. Mater.* **80**, 211-218 (1989).
